# Supplementary material for: Meiotic DNA break resection and recombination rely on chromatin remodeler Fun30
Source: EMBO J. 2024 Nov 29;44(1):200–24. doi: 10.1038/s44318-024-00318-8 (PMC11695836; doi:10.1038/s44318-024-00318-8)
Supplement: Supplementary file 1 — Appendix [file 44318_2024_318_MOESM1_ESM.pdf]

Appendix

Meiotic DNA break resection and recombination rely on chromatin remodeler Fun30

Pei-Ching Huang, Soogil Hong, Hasan F. Alnaser, Eleni P. Mimitou, Keun P. Kim, Hajime Murakami, and Scott Keeney

Table of contents:

Appendix Figure S1 Page 2
Appendix Figure S2 Page 4
Appendix Table S1 Page 5
Appendix Table S2 Page 7
Appendix Table S3 Page 8
Appendix Table S4 Page 9

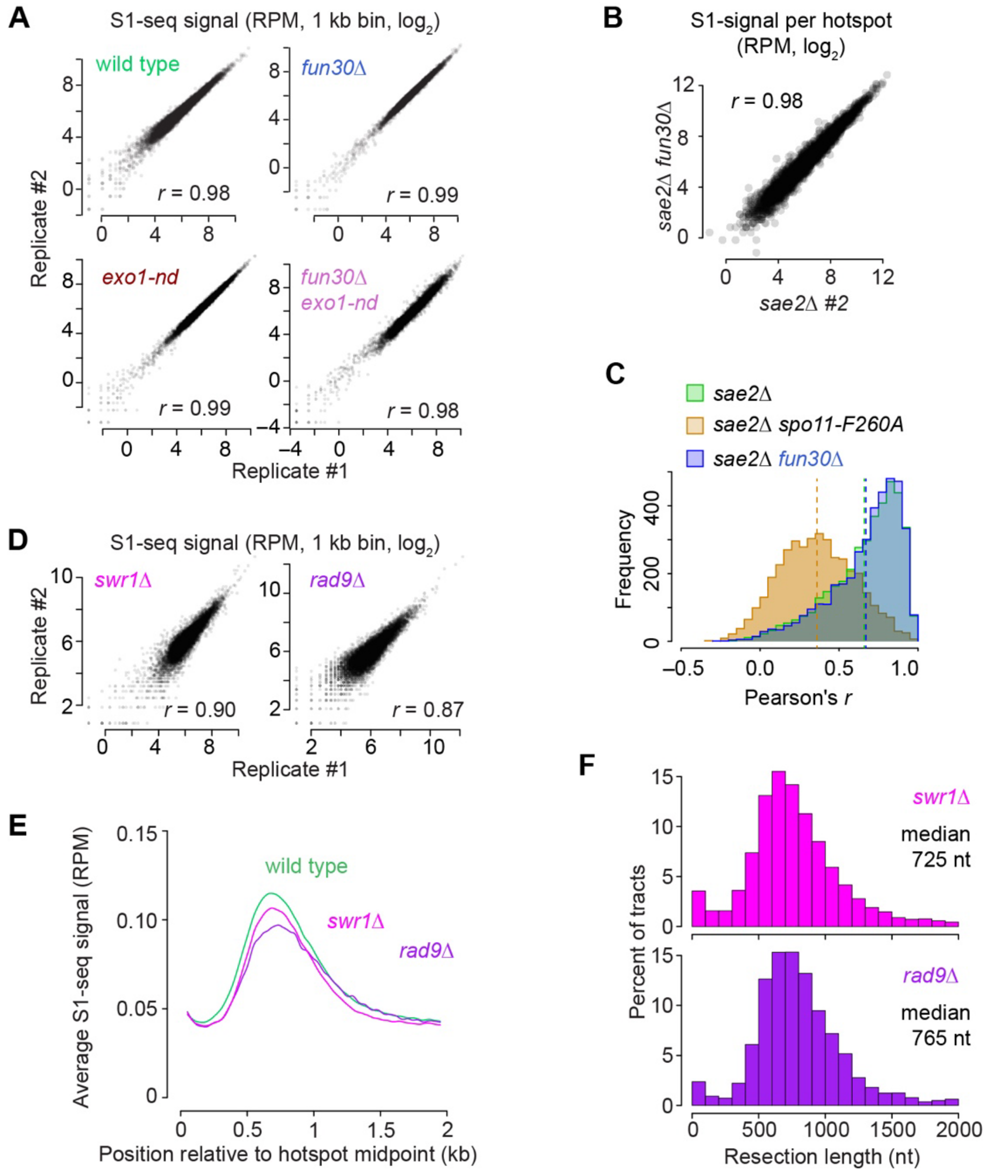

Appendix Figure S1. S1-seq reproducibility and S1-seq in *swr1*Δ and *rad9*Δ

(A) Reproducibility of S1-seq between two biological replicates of each genotype. Each point is the $\log_2$-transformed read count in a 1-kb segment of the genome.

(B) Preservation of hotspot heats in *fun30*Δ. Each dot represents the sum of the S1-seq signal at a given hotspot in a *sae2*Δ background (n = 3908). S1-seq library preparation was previously shown to quantitatively capture the unresected DSBs that accumulate in *sae2*Δ mutants (Mimitou et al. 2017; Mimitou and Keeney 2018).

(C) Preservation of fine-scale DSB distributions within hotspots in *fun30*Δ. For each of 3908 DSB hotspots, we computed the correlation coefficient (Pearson's *r*) comparing the S1-seq spatial distribution between the indicated pairs of datasets, then plotted the distributions of the 3908 correlation coefficients. The vertical dashed lines indicate the mean *r* value for each comparison. We compared two biological replicates of *sae2*Δ to show the intrinsic variability in this measurement (mean *r* = 0.66) and we compared *sae2*Δ to *sae2*Δ *spo11-F260A* (Claeys Bouuaert et al. 2021) as an example of what happens in a mutant with altered DSB distributions (mean *r* = 0.36). Comparison of *sae2*Δ with *fun30*Δ *sae2*Δ gave a distribution of correlation coefficients indistinguishable from the comparison of *sae2*Δ replicates (mean *r* = 0.67), indicating that the *fun30*Δ mutation has little or no measurable effect on local DSB distributions within hotspots.

(D) Reproducibility of *swr1*Δ and *rad9*Δ S1-seq biological replicates.
(E) Average S1-seq distribution around hotspots in *swr1*Δ and *rad9*Δ*.*
(F) Distribution of resection tract lengths in *swr1*Δ and *rad9*Δ as in **Figure 2C**.

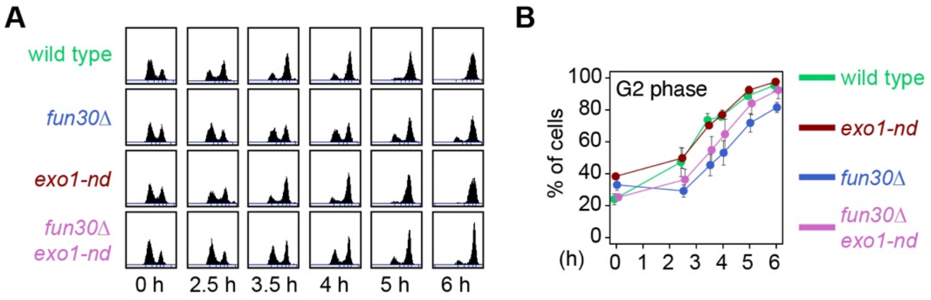

Appendix Figure S2. Analysis of early meiotic progression by flow cytometric DNA analysis.
(A) Representative histograms of flow cytometric measurements of the cellular DNA content.
(B) Progression of pre-meiotic DNA replication during early meiosis. The percentage of cells in G2 phase was calculated from flow cytometry (mean ± SD for three independent meiotic cultures).

Appendix Table S1. List of yeast strains used in this study.

S1-seq strains	strains	MAT	genotypes[†]
Wild type	SKY6066	a/α	nuc1Δ::LEU2/"
exo1-nd	SKY6075	a/α	nuc1Δ::LEU2/", exo1(D173A)/"
fun30Δ	SKY6457	a/α	nuc1Δ::LEU2/", fun30Δ::KanMX/"
fun30Δ exo1-nd	SKY6593	a/α	nuc1Δ::LEU2/", fun30Δ::KanMX/", exo1(D173A)/"
sae2Δ	SK 6436	a/α	nuc1Δ::LEU2/", sae2Δ::NatMX/"
fun30Δ sae2Δ	SKY6587	a/α	nuc1Δ::LEU2/", fun30Δ::KanMX/", sae2Δ::NatMX/"
**S1 Southern and S1-seq strains**	**strains**	**MAT**	**genotypes[†]**
Wild type	SKY6066	a/α	nuc1Δ::LEU2/"
sae2Δ	SKY6436	a/α	nuc1Δ::LEU2/", sae2Δ::NatMX/"
exo1-nd	SKY6075	a/α	nuc1Δ::LEU2/", exo1(D173A)/"
arp8Δ	SKY6094	a/α	nuc1Δ::LEU2/", arp8Δ::KanMX4/"
htz1Δ	SKY6081	a/α	nuc1Δ::LEU2/", htz1Δ::KanMX/"
swr1Δ	SKY6430	a/α	nuc1Δ::LEU2/", swr1Δ::KanMX/"
fun30Δ	SKY6457	a/α	nuc1Δ::LEU2/", fun30Δ::KanMX/"
fun30Δ sae2Δ	SKY6587	a/α	nuc1Δ::LEU2/", fun30Δ::KanMX/", sae2Δ::NatMX/"
fun30Δ exo1-nd	SKY6593	a/α	nuc1Δ::LEU2/", fun30Δ::KanMX/", exo1(D173A)/"
fun30k603R	SKY6736	a/α	nuc1Δ::LEU2/", fun30K603R::URA3/", fun30Δ::KanMX/"
arp8Δ htz1Δ	SKY6424	a/α	nuc1Δ::LEU2/", arp8Δ::KanMX/", htz1Δ::KanMX4/"
arp8Δ swr1Δ	SKY6504	a/α	nuc1Δ::LEU2/", arp8Δ::KanMX/", swr1Δ::KanMX4/"
arp8Δ fun30Δ	SKY6498	a/α	nuc1Δ::LEU2/", arp8Δ::KanMX/", fun30Δ::KanMX4/"
htz1Δ swr1Δ	SKY6492	a/α	nuc1Δ::LEU2/", htz1Δ::KanMX/", swr1Δ::KanMX4/"
htz1Δ fun30Δ	SKY6486	a/α	nuc1Δ::LEU2/", htz1Δ::KanMX/", fun30Δ::KanMX4/"
swr1Δ fun30Δ	SKY6510	a/α	nuc1Δ::LEU2/", swr1Δ::KanMX/", fun30Δ::KanMX4/"
**Flourescent spore assay (FSA)**	**strains**	**MAT**	**genotypes[†]**
Wild type	HMY339	a/α	trp1::hisG/", CEN8::tdTomato-LEU2/CEN8; ARG4::GFP*-URA3/ARG4; THR1/THR1::m-Cerulean-TRP1
exo1-nd	HMY859	a/α	trp1::hisG/", CEN8::tdTomato-LEU2/CEN8; ARG4::GFP*-URA3/ARG4; THR1/THR1::m-Cerulean-TRP1, exo1(D173A)/"
fun30Δ	HMY856	a/α	trp1::hisG/", CEN8::tdTomato-LEU2/CEN8; ARG4::GFP*-URA3/ARG4; THR1/THR1::m-Cerulean-TRP1, fun30Δ::KanMX4/"
fun30Δ exo1-nd	HMY858	a/α	trp1::hisG/", CEN8::tdTomato-LEU2/CEN8; ARG4::GFP*-URA3/ARG4; THR1/THR1::m-Cerulean-TRP1, exo1(D173A)/", fun30Δ::KanMX4/"
**ChIP-seq**	**strains**	**MAT**	**genotypes[†]**
untagged	SKY6066	a/α	nuc1Δ::LEU2/"
Fun30-myc sae2Δ	SKY6822	a/α	sae2Δ::NatMX/", nuc1Δ::LEU2/", Fun30-myc-KanMX/"
Fun30-myc spo11-yf sae2Δ	SKY7231	a/α	spo11yf::KanMX4/", sae2Δ::NatMX/", nuc1Δ::LEU2/", Fun30-myc-KanMX/"
Myc-tagged Rec114 (S.mikatae IFO1815): spike in	SKY6052	a/α	REC114-myc8::ura3::HphMX6/REC114-myc8::ura3::HphMX6
**Physical analysis**	**strains**	**MAT**	**genotypes[‡]**
Wild type	KKY2945	a/α	HIS4::LEU2-(BamHI)/his4x::LEU2-(NgoMIV)--URA3, nuc1Δ::LEU2/", ERG1::SalI/ERG1::SpeI
fun30Δ	KKY6069	a/α	HIS4::LEU2-(BamHI)/his4x::LEU2-(NgoMIV)--URA3, nuc1Δ::LEU2/", ERG1::SalI/ERG1::SpeI, fun30Δ::KanMX4/"
exo1-nd	KKY6101	a/α	HIS4::LEU2-(BamHI)/his4x::LEU2-(NgoMIV)--URA3, nuc1Δ::LEU2/", ERG1::SalI/ERG1::SpeI, exo1(D173A)/"
fun30Δ exo1-nd	KKY4555	a/α	HIS4::LEU2-(BamHI)/his4x::LEU2-(NgoMIV)--URA3, nuc1Δ::LEU2/", ERG1::SalI/ERG1::SpeI, fun30::KanMX4/", exo1(D173A)/"

All strains except SKY6052 are isogenic derivatives of SK1 background.
† All strains are also homozygous for the mutation *ho::LYS2, lys2, ura3, leu2::hisG.*
‡ All strains are also homozygous for the mutation *ho::hisG*, *leu2::hisG*, *ura3 (ΔPstI-SmaI)*.

Appendix Table S2. Genetic distance and MI nondisjunction estimated by fluorescent spore assay

		wild type	*exo1-nd*	*fun30Δ*	*fun30Δ exo1-nd*
CEN8-ARG4	PD:TT:NPD	882:358:0	1293:347:2	1393:514:3	1604:391:4
	cM ± SE	14.44 ± 0.64	10.93 ± 0.56	13.93 ± 0.57	10.38 ± 0.53
	p (vs wild type)	N/A	$4.1x10^{-6}$	0.11	$1.9x10^{-9}$
ARG4-THR1	PD:TT:NPD	1143:97:0	1482:160:0	1662:248:0	1807:192:0
	cM ± SE	3.91 ± 0.38	4.87 ± 0.37	6.49 ± 0.38	4.80 ± 0.33
	p (vs wild type)	N/A	0.20	$2.3x10^{-5}$	0.22
CEN8-THR1	PD:TT:NPD	792:448:0	1142:497:3	1161:745:4	1439:553:7
	cM ± SE	18.06 ± 0.68	15.68 ± 0.64	20.13 ± 0.63	14.88 ± 0.63
	p (vs wild type)	N/A	$8.5x10^{-4}$	0.033	$1.4x10^{-7}$
	MI NDJ	1/1241 = 0.081%	2/1644 = 0.12%	3/1913 = 0.16%	54/2053 = 2.6%
	p (vs wild type)	N/A	0.38	0.20	$2.8x10^{-61}$

The number of tetrads exhibiting fluorescent marker configurations in parental ditype (PD), tetratype (TT) and nonparental ditype (NPD) are shown (see Source Data Table S2 for raw data). Genetic distances were calculated using Perkins equation [cM = 100*(6*NPD+TT)/(2*(PD+NPD+TT))]. Standard errors (SE) were calculated using Stahl Lab Online Tools (https://elizabethhousworth.com/StahlLabOnlineTools/).

Appendix Table S3. Raw data for fluorescent spore assay

Tetrad type	[a]Marker configuration	wild type	*exo1-nd*	*fun30∆*	*fun30∆ exo1-nd*
1. parental	RG, RG, C, C	790	1139	1154	1431
2. single CO at *CEN8-ARG4*	RG, RC, G, C	353	341	505	372
3. single CO at *ARG4-THR1*	RGC, RG, C, E	92	154	239	173
4. double CO type i	RG, RC, GC, E	0	0	1	1
5. double CO type ii	RGC, RC, G, E	0	1	1	3
6. double CO type iii	RGC, R, G, C	3	2	0	7
7. double CO type iv	RG, R, GC, C	2	3	7	8
8. NPD at *CEN8-ARG4*	RC, RC, G, G	0	2	3	4
9. MI NDJ	RGC, RGC, E, E	1	2	3	54
Total		1241	1644	1913	2053

The numbers of scored tetrads exhibiting indicated fluorescent marker configurations are shown. The tetrads type 9 were scored as MI NDJ and excluded from genetic distance estimation (Thacker et al. 2011). For each genetic interval, the numbers of tetrads with PD, TT, and NPD configurations were calculated using the following formula:

CEN8-ARG4: PD = types 1+3, TT = types 2+4+5+6+7, NPD = type 8

ARG4-THR1: PD = types 1+2+8, TT = types 3+4+5+6+7, NPD = N/A (set as 0)

CEN8-THR1: PD = types 1+7, TT = types 2+3+4+6, NPD = types 5+8

[a]Letters indicate fluorescent marker signals of spores in the tetrad. R: RFP, G: GFP, C: CFP, E: empty (no signal).

Appendix Table S4. Summary of genomic datasets

S1-seq data (GSE221377)

s_sky6066_4h_index1_HITMAP	wild type
s_sky6066_4h_index2_HITMAP	wild type
s_sky6075_4h_index5_HITMAP	*exo1-nd*
s_sky6075_4h_index6_HITMAP	*exo1-nd*
s_sky6057_4h_index1_HITMAP	*fun30Δ*
s_sky6057_4h_index2_HITMAP	*fun30Δ*
s_sky6593_4h_index3_HITMAP	*fun30Δ exo1-nd*
s_sky6593_4h_index4_HITMAP	*fun30Δ exo1-nd*
s_SKY6436_4h_1_HITMAP	*sae2Δ*
s_SKY6587_4h_1_HITMAP	*fun30Δ sae2Δ*
s_SKY6587_4h_2_HITMAP	*fun30Δ sae2Δ*

ChIP-seq data (GSE221033)

s_sky6066-1input	untagged
s_sky6066-2input	untagged
s_sky6822-1input	*Fun30myc sae2Δ*
s_sky6822-2input	*Fun30myc sae2Δ*
s_sky7231-1input	*Fun30myc spo11yf sae2Δ*
s_sky7231-2input	*Fun30myc spo11yf sae2Δ*
s_sky6066-1IP	untagged
s_sky6066-2IP	untagged
s_sky6822-1IP	*Fun30myc sae2Δ*
s_sky6822-2IP	*Fun30myc sae2Δ*
s_sky7231-1IP	*Fun30myc spo11yf sae2Δ*
s_sky7231-2IP	*Fun30myc spo11yf sae2Δ*
