# Supplementary figures and images for: Meiotic DNA break resection and recombination rely on chromatin remodeler Fun30
Source: EMBO J. 2024 Nov 29;44(1):200–24. doi: 10.1038/s44318-024-00318-8 (PMC11695836; doi:10.1038/s44318-024-00318-8)

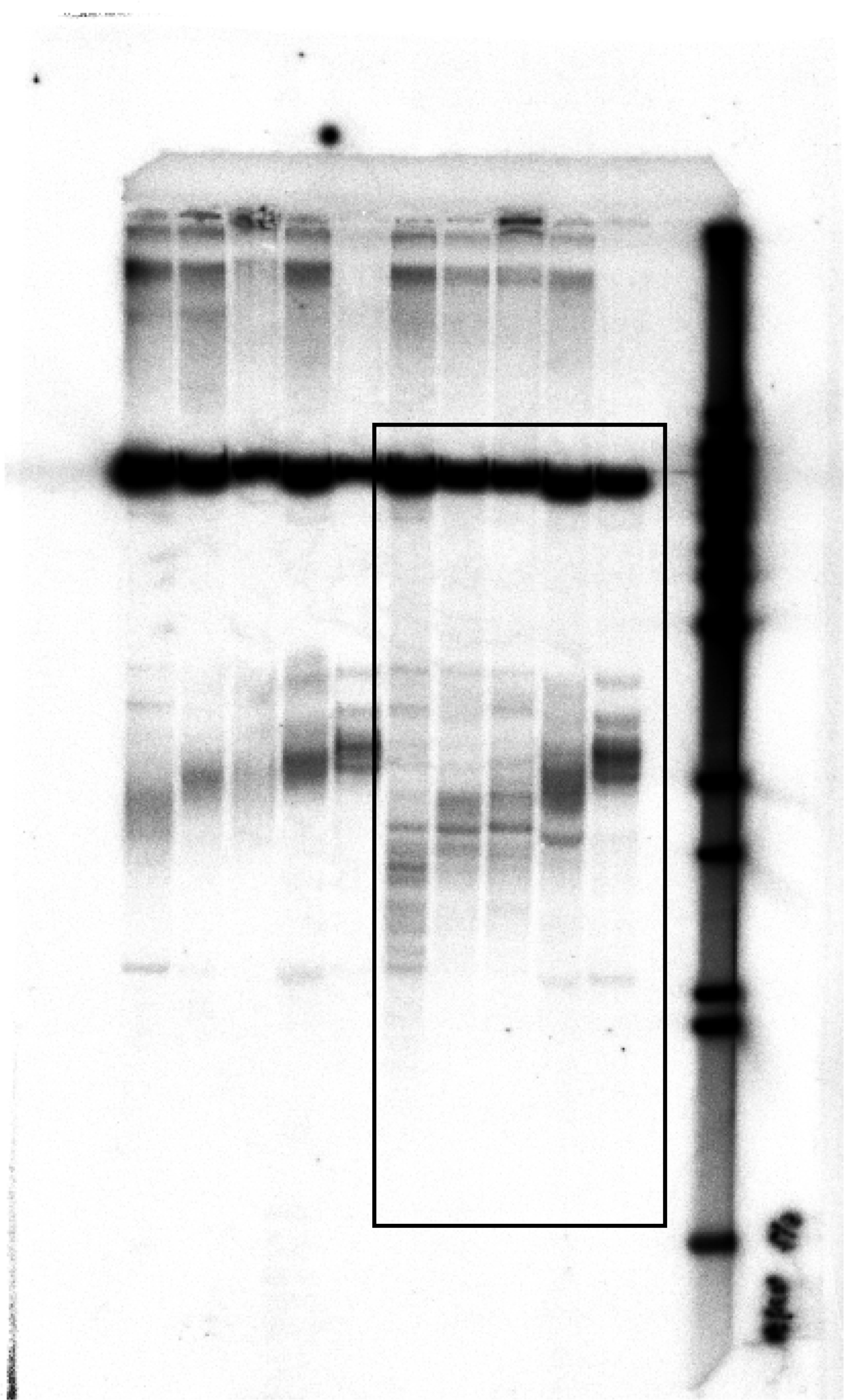

Supplement: Supplementary file 3 — Source data Fig. 1 [file 44318_2024_318_MOESM3_ESM.zip › SD Fig 1/1B/GAT1iii.tif]

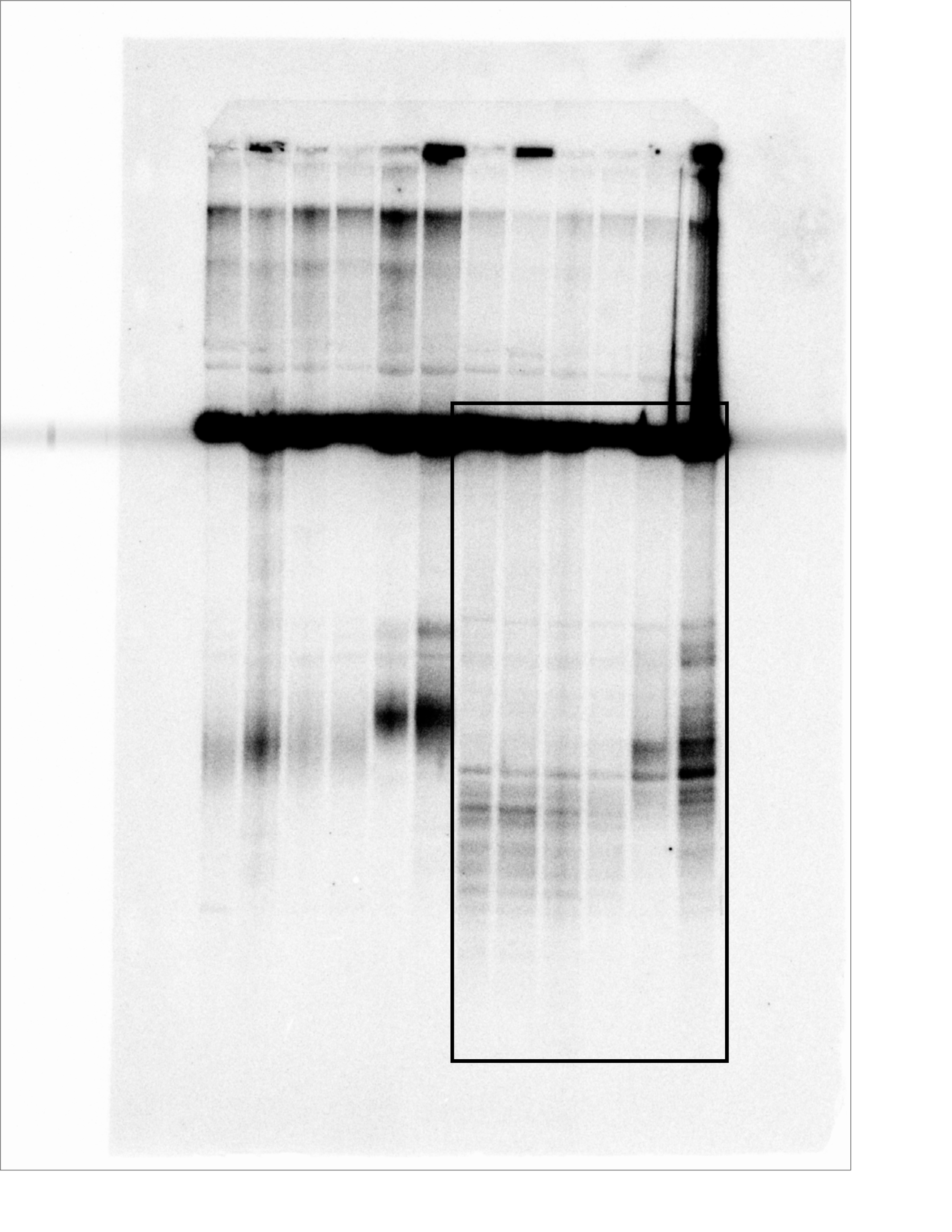

Supplement: Supplementary file 3 — Source data Fig. 1 [file 44318_2024_318_MOESM3_ESM.zip › SD Fig 1/1B/GAT1i.tif]

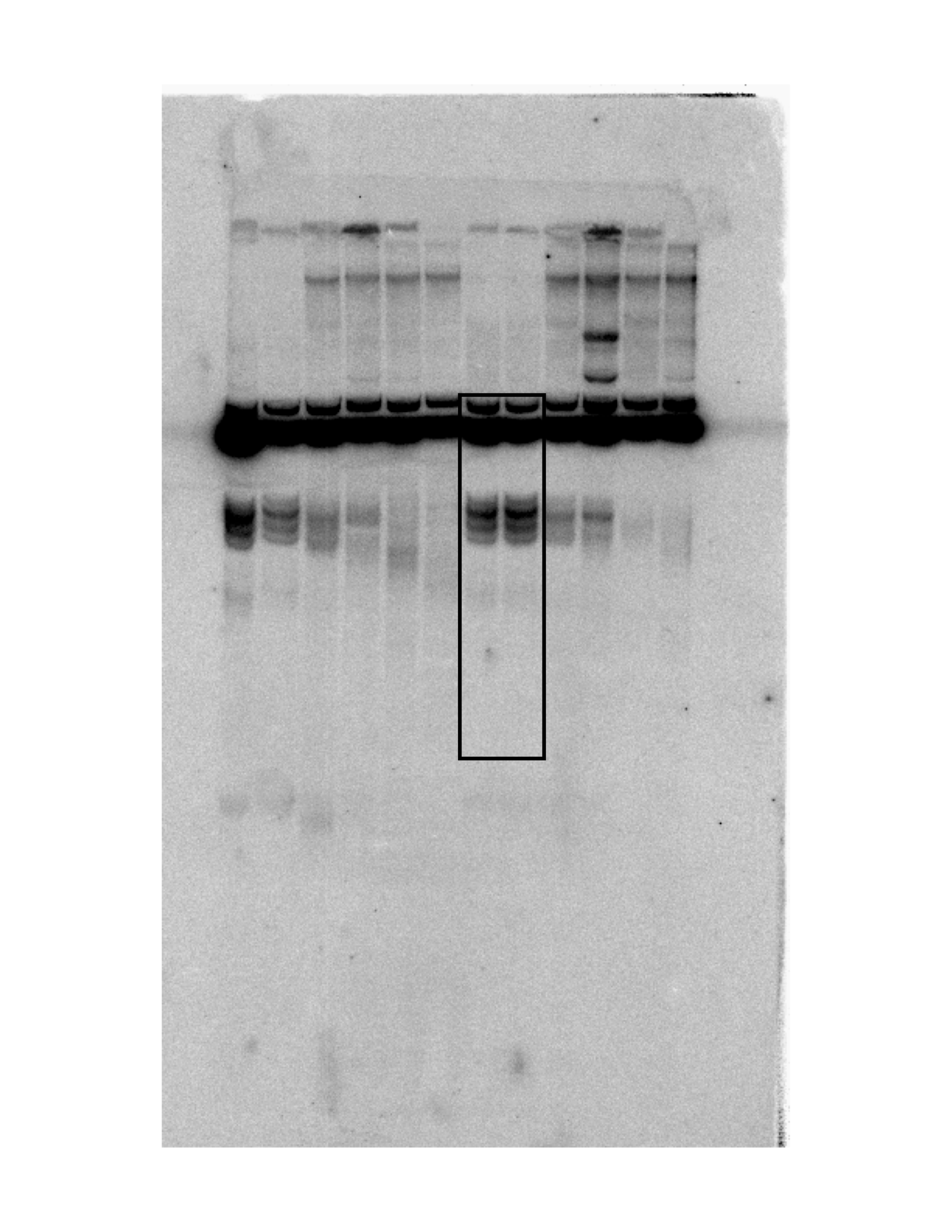

Supplement: Supplementary file 3 — Source data Fig. 1 [file 44318_2024_318_MOESM3_ESM.zip › SD Fig 1/1B/CCT6iv.tif]

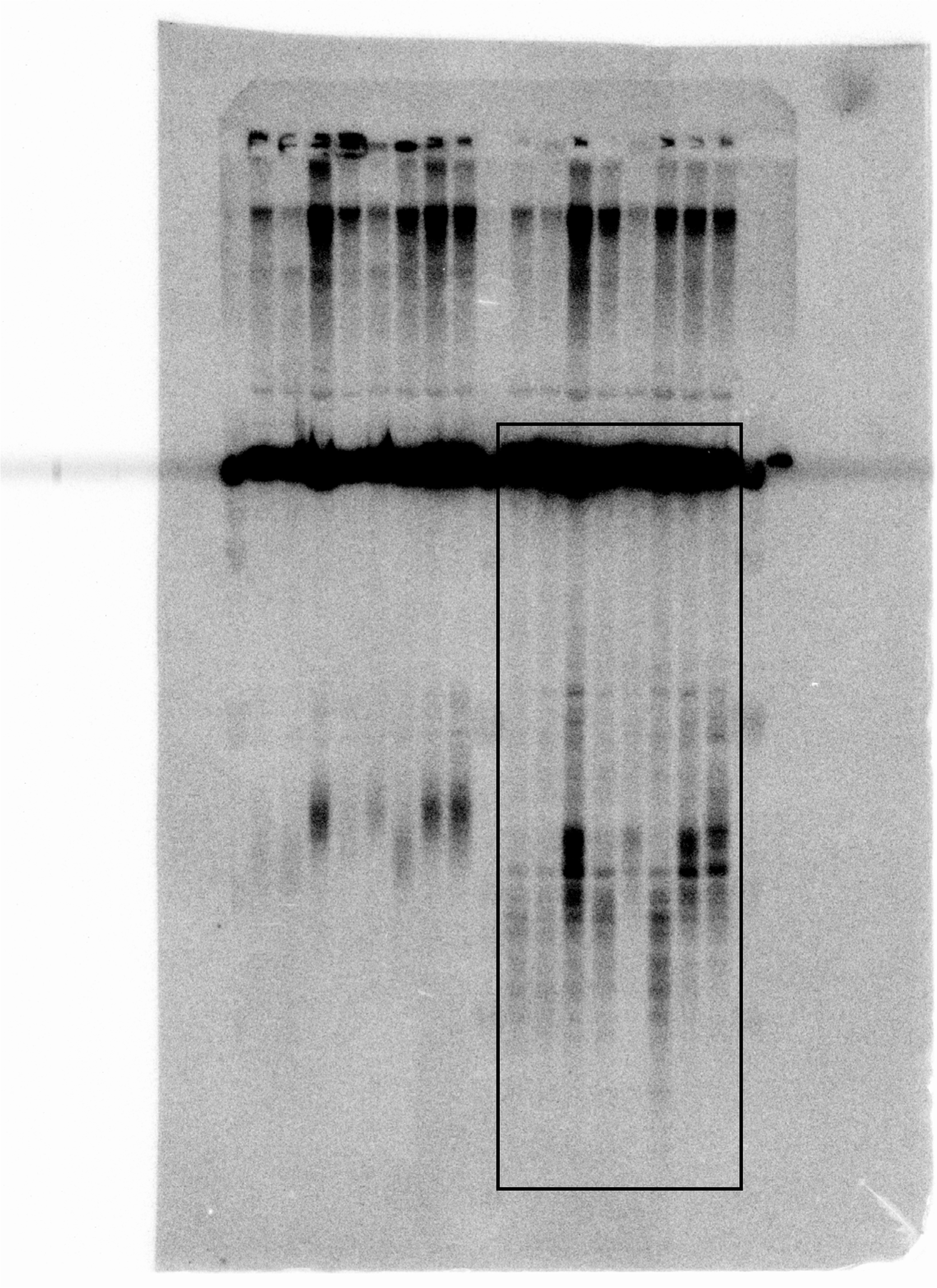

Supplement: Supplementary file 3 — Source data Fig. 1 [file 44318_2024_318_MOESM3_ESM.zip › SD Fig 1/1B/GAT1ii.tif]

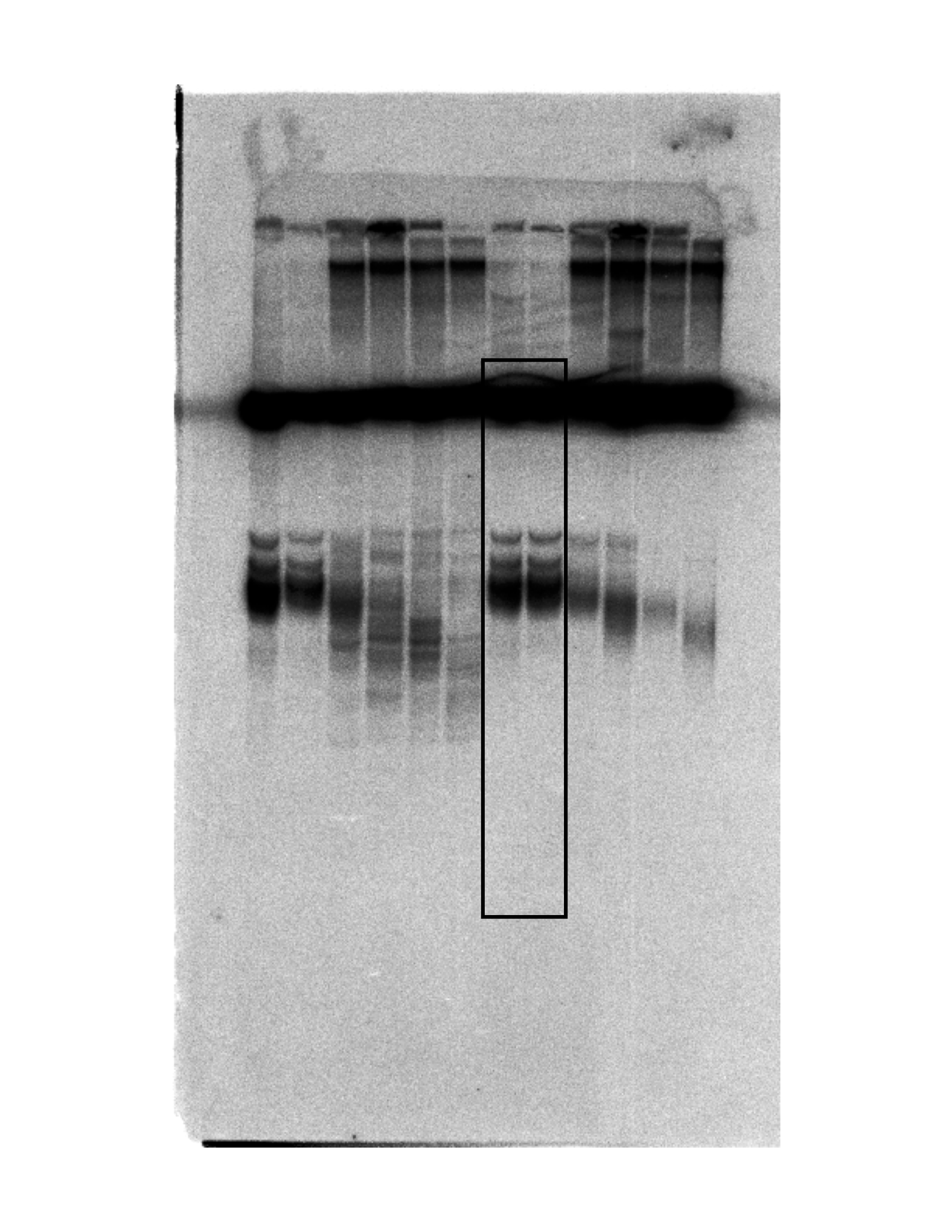

Supplement: Supplementary file 3 — Source data Fig. 1 [file 44318_2024_318_MOESM3_ESM.zip › SD Fig 1/1B/GAT1iv.tif]

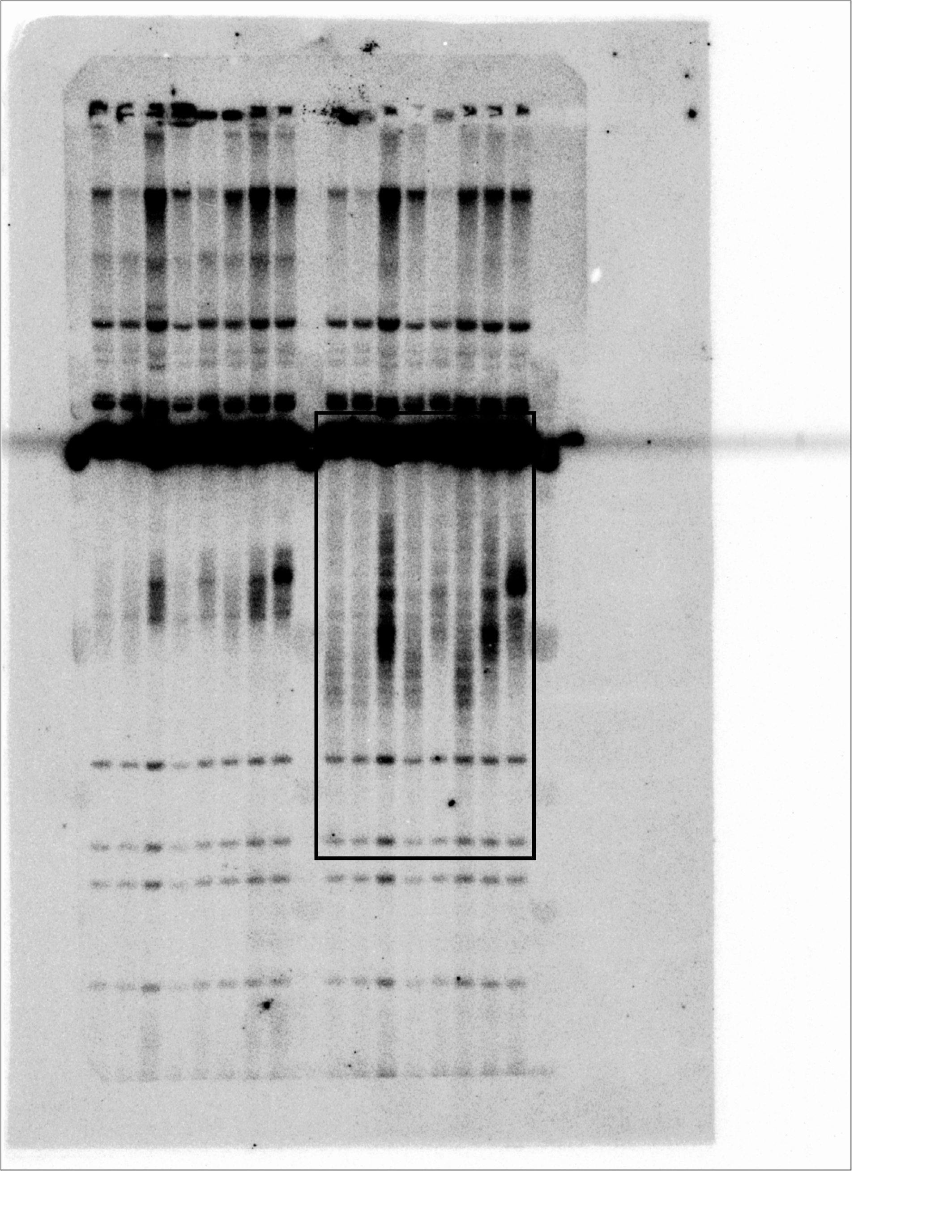

Supplement: Supplementary file 3 — Source data Fig. 1 [file 44318_2024_318_MOESM3_ESM.zip › SD Fig 1/1B/CCT6ii.tif]

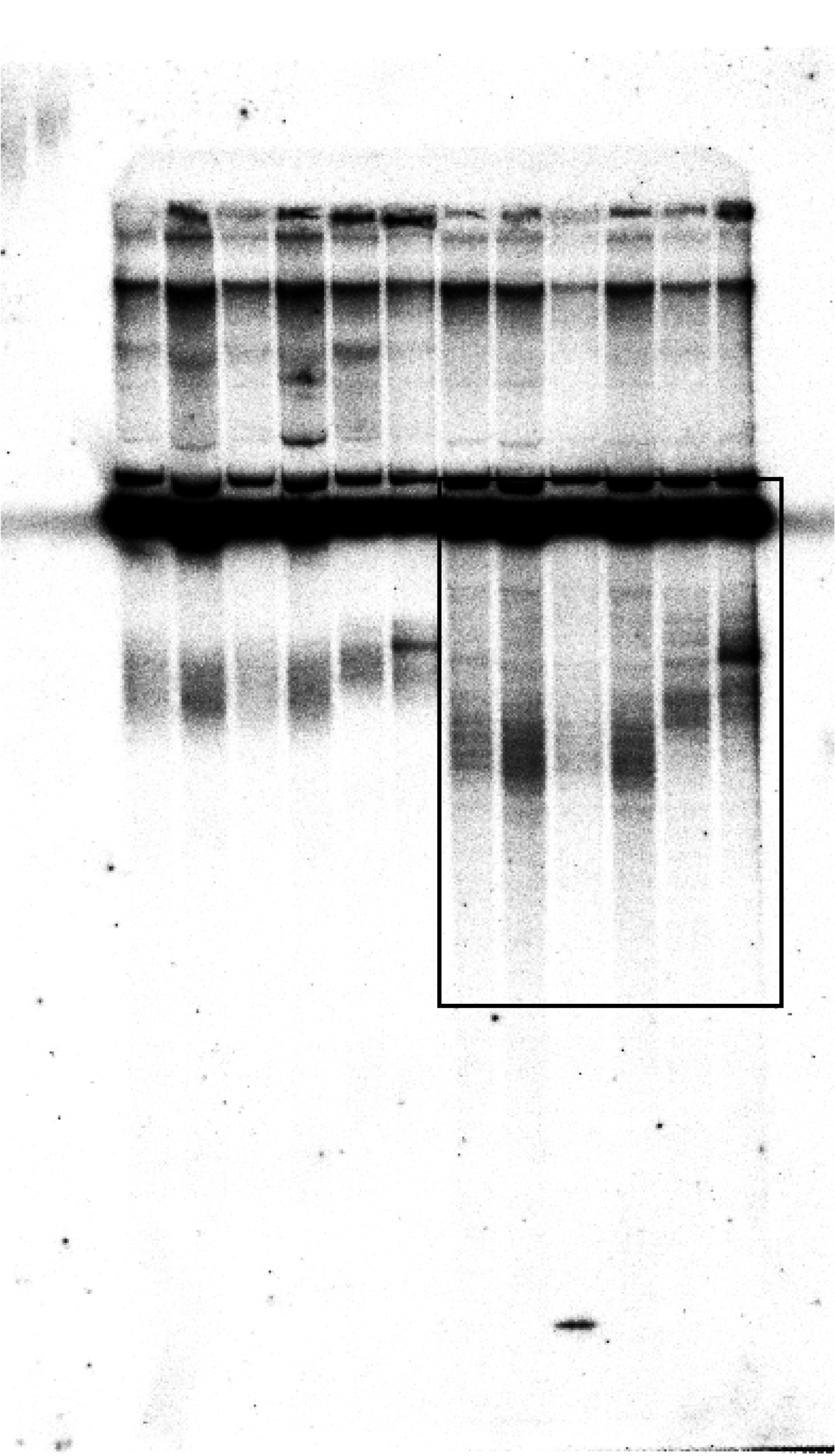

Supplement: Supplementary file 3 — Source data Fig. 1 [file 44318_2024_318_MOESM3_ESM.zip › SD Fig 1/1B/CCT6i.tif]

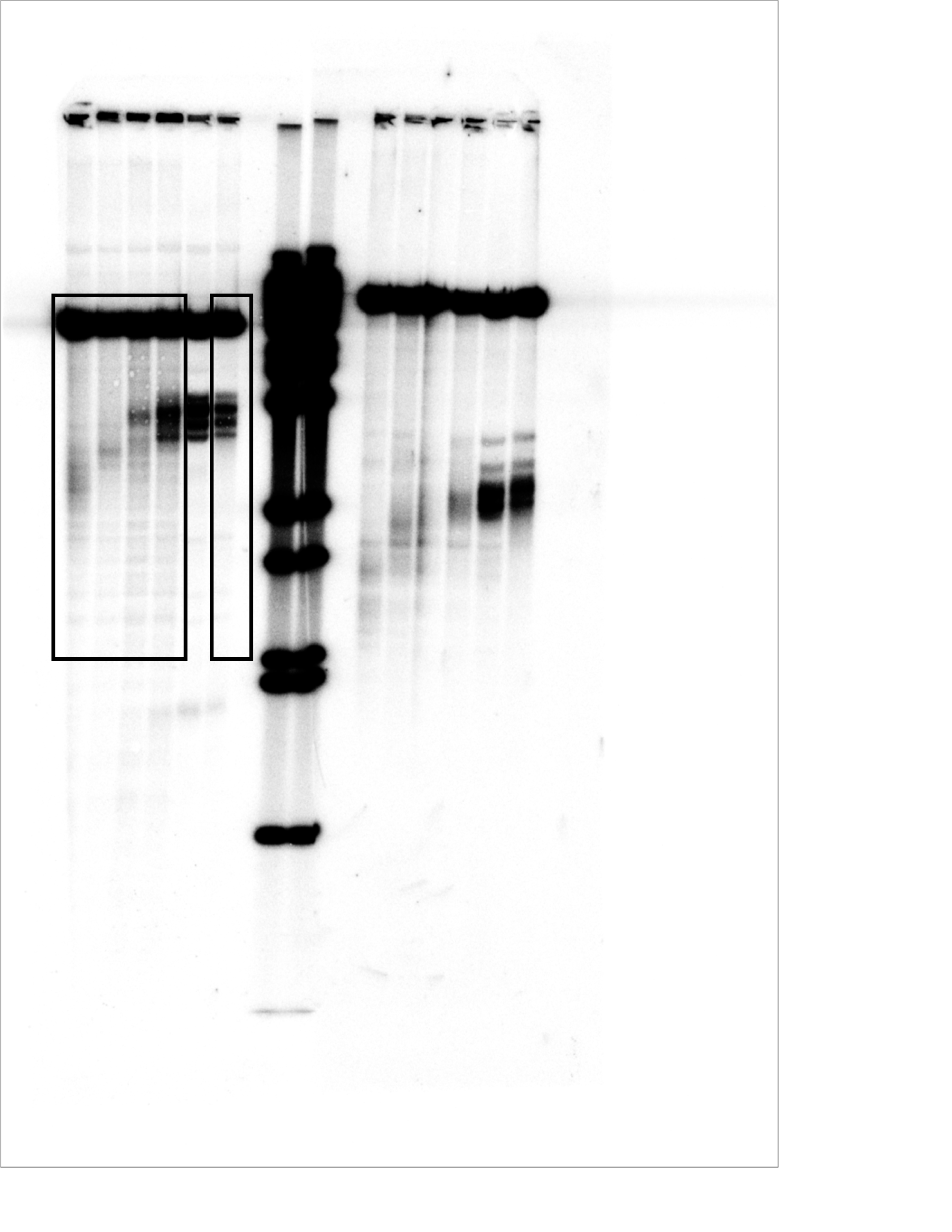

Supplement: Supplementary file 3 — Source data Fig. 1 [file 44318_2024_318_MOESM3_ESM.zip › SD Fig 1/1B/CCT6iii.tif]

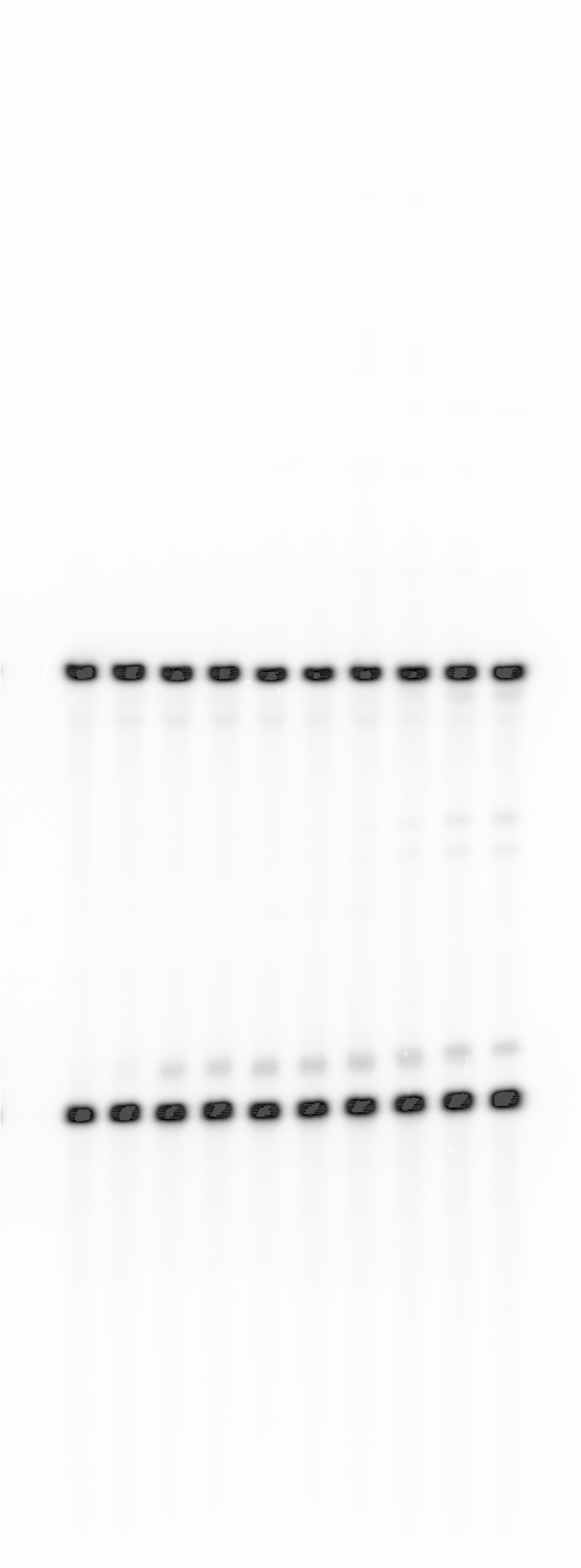

Supplement: Supplementary file 5 — Source data Fig. 6 [file 44318_2024_318_MOESM5_ESM.zip › SD Fig 6/6F/fun30D exo1-nd_CO,NCO gel.tif]

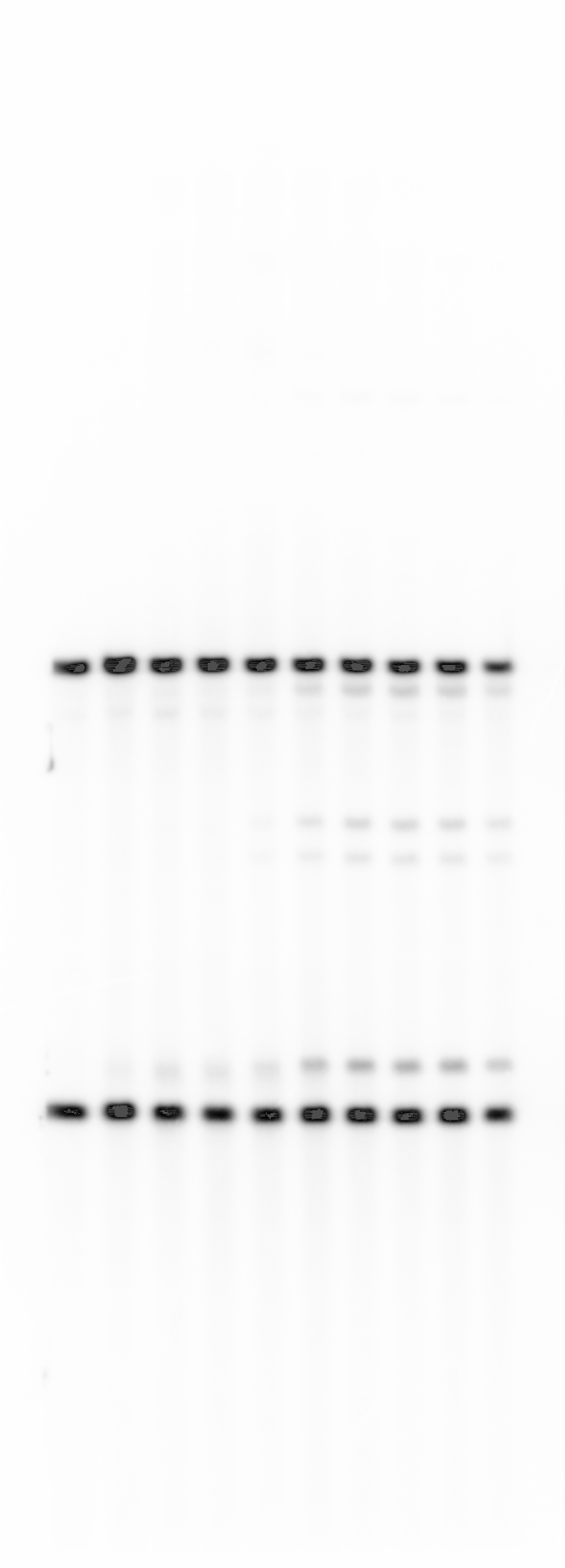

Supplement: Supplementary file 5 — Source data Fig. 6 [file 44318_2024_318_MOESM5_ESM.zip › SD Fig 6/6F/exo1-nd_CO,NCO gel.tif]

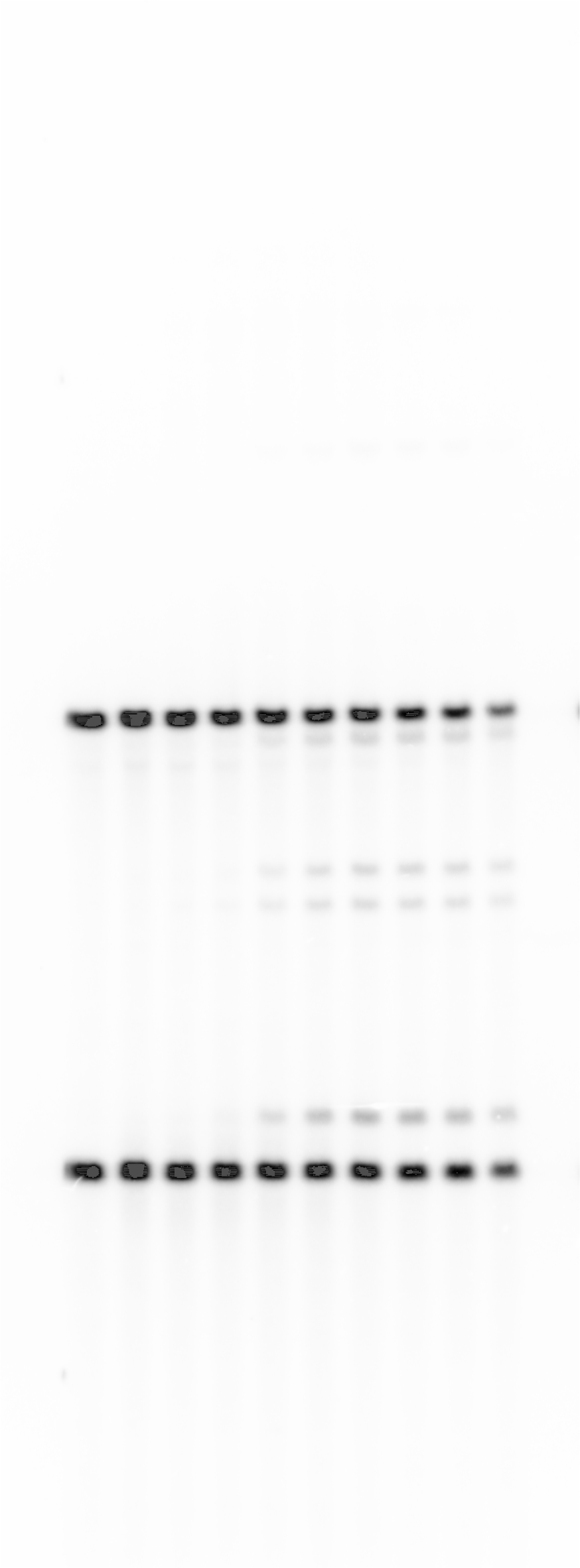

Supplement: Supplementary file 5 — Source data Fig. 6 [file 44318_2024_318_MOESM5_ESM.zip › SD Fig 6/6F/WT_CO,NCO gel.tif]

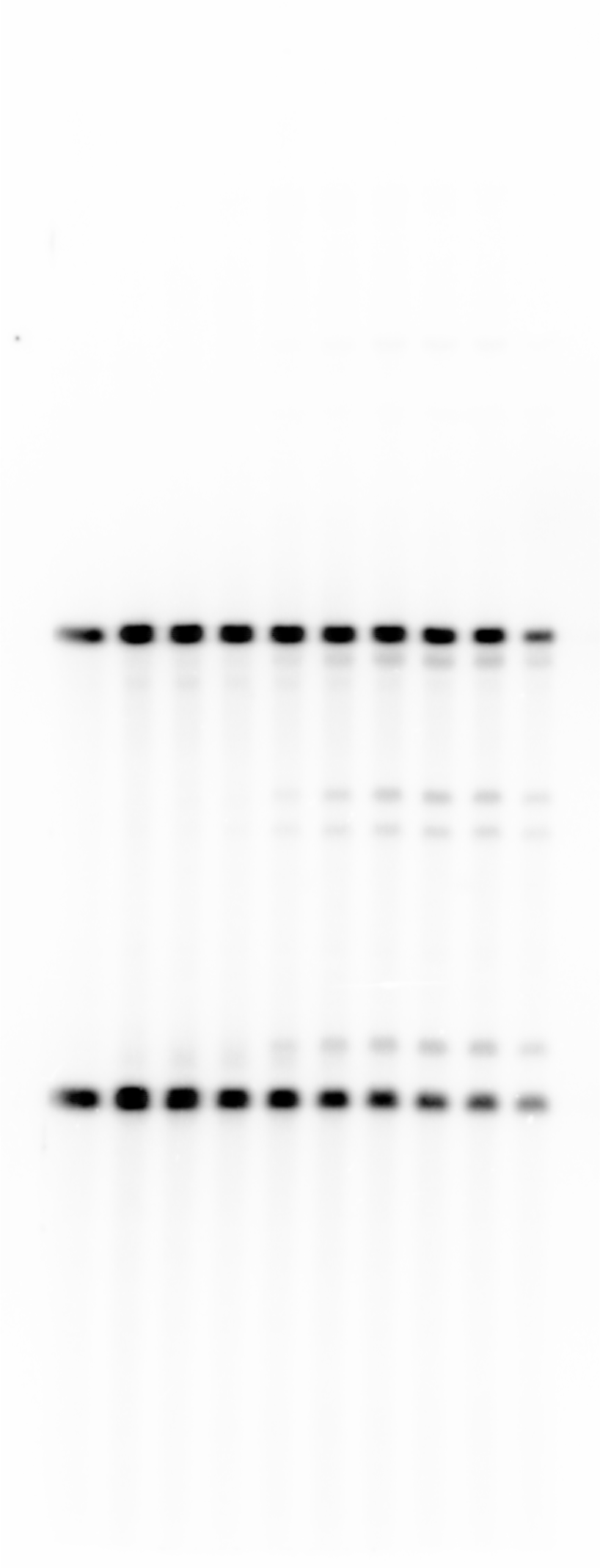

Supplement: Supplementary file 5 — Source data Fig. 6 [file 44318_2024_318_MOESM5_ESM.zip › SD Fig 6/6F/fun30D_CO,NCO gel.tif]

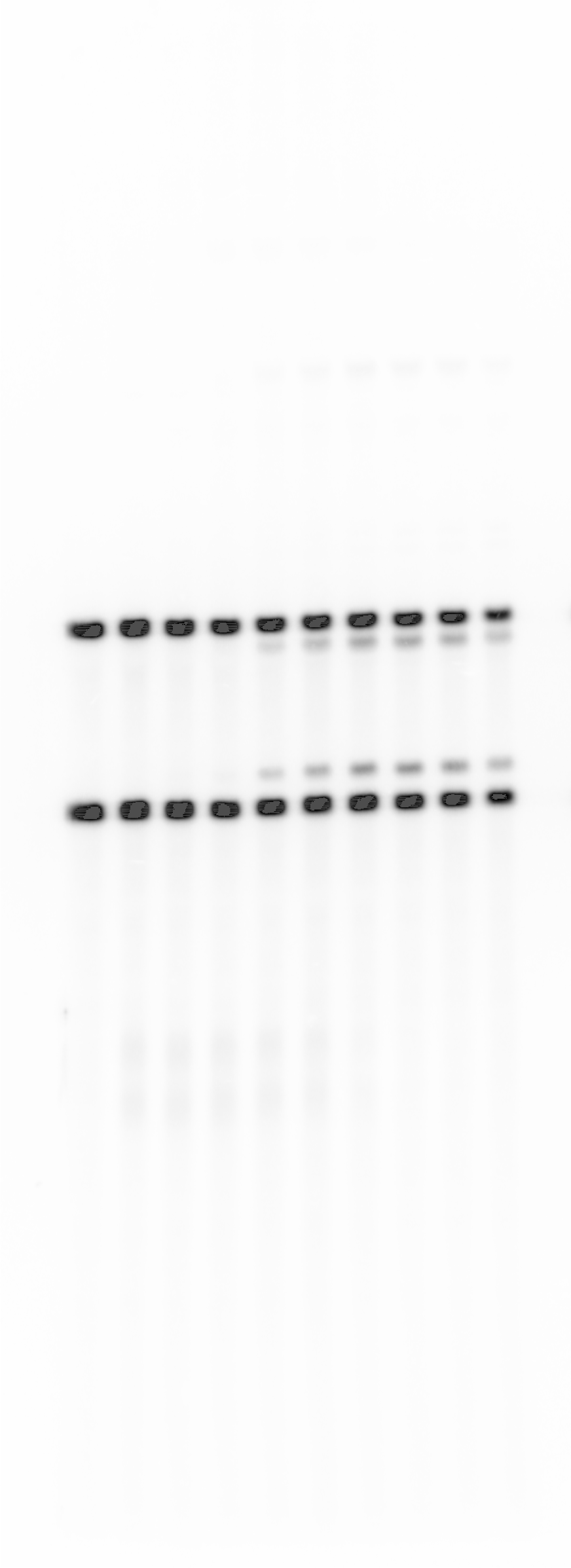

Supplement: Supplementary file 5 — Source data Fig. 6 [file 44318_2024_318_MOESM5_ESM.zip › SD Fig 6/6A/WT_1D gel.tif]

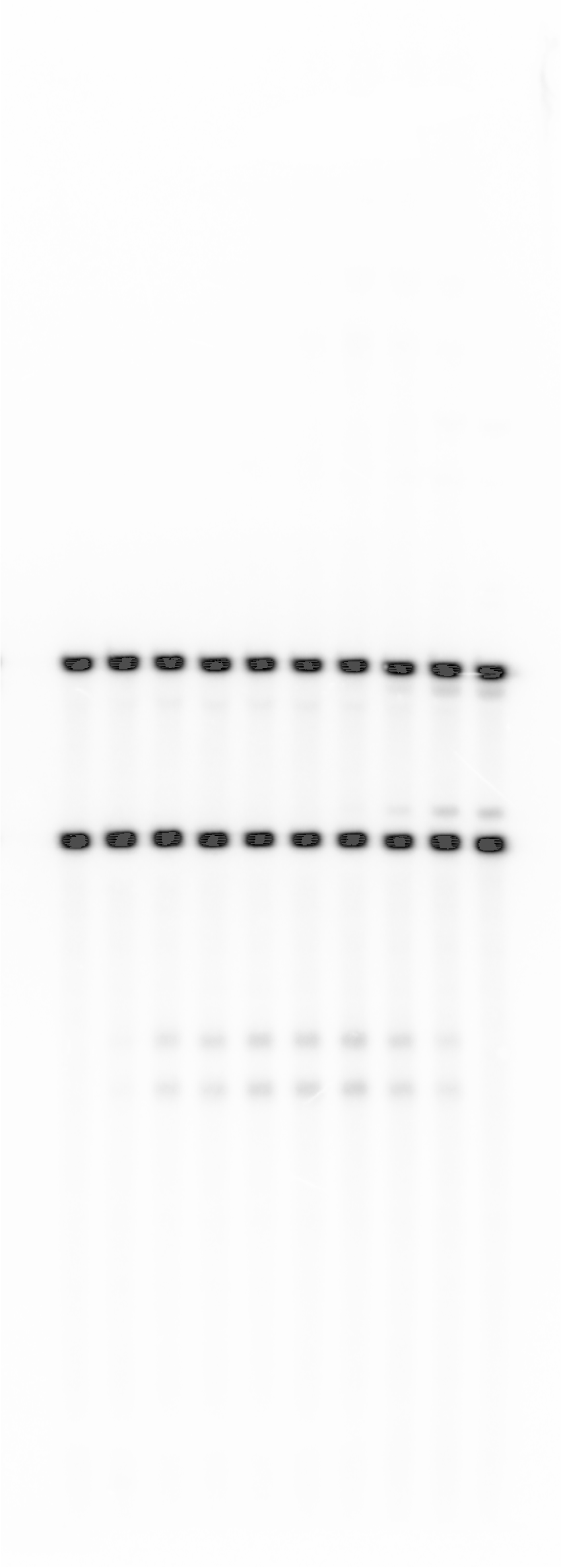

Supplement: Supplementary file 5 — Source data Fig. 6 [file 44318_2024_318_MOESM5_ESM.zip › SD Fig 6/6A/fun30D exo1-nd_1D gel.tif]

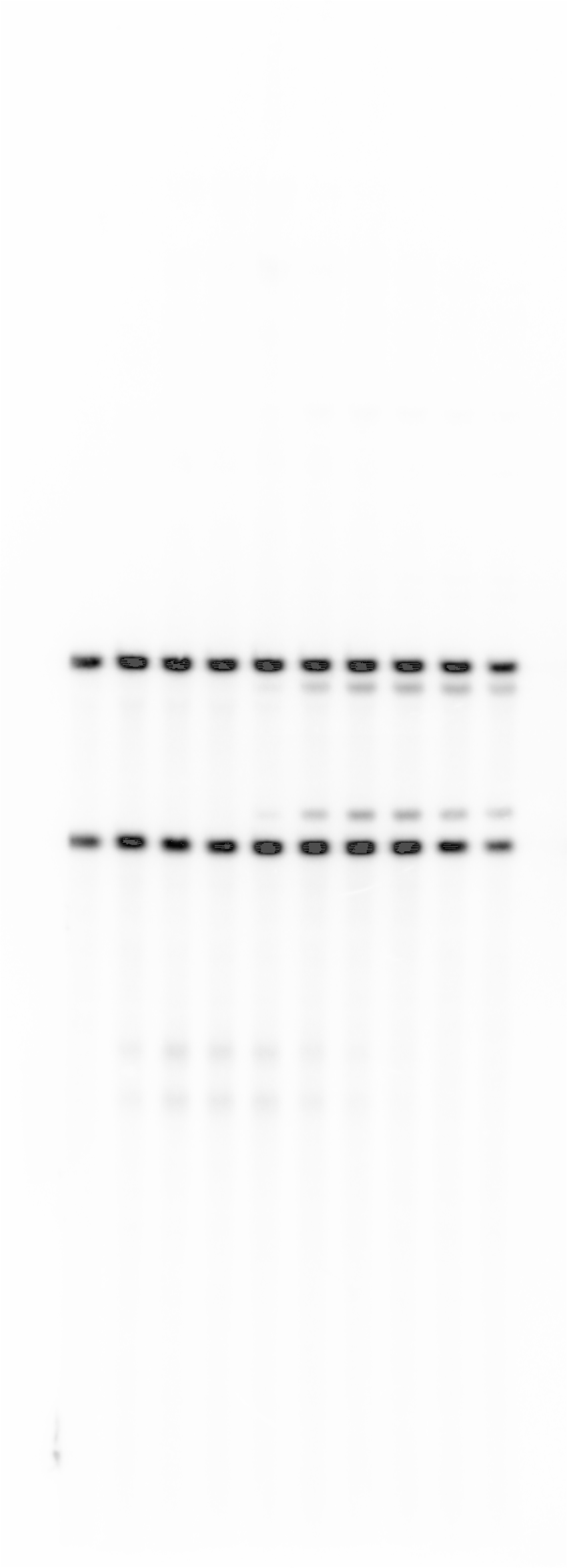

Supplement: Supplementary file 5 — Source data Fig. 6 [file 44318_2024_318_MOESM5_ESM.zip › SD Fig 6/6A/exo1-nd_1D gel.tif]

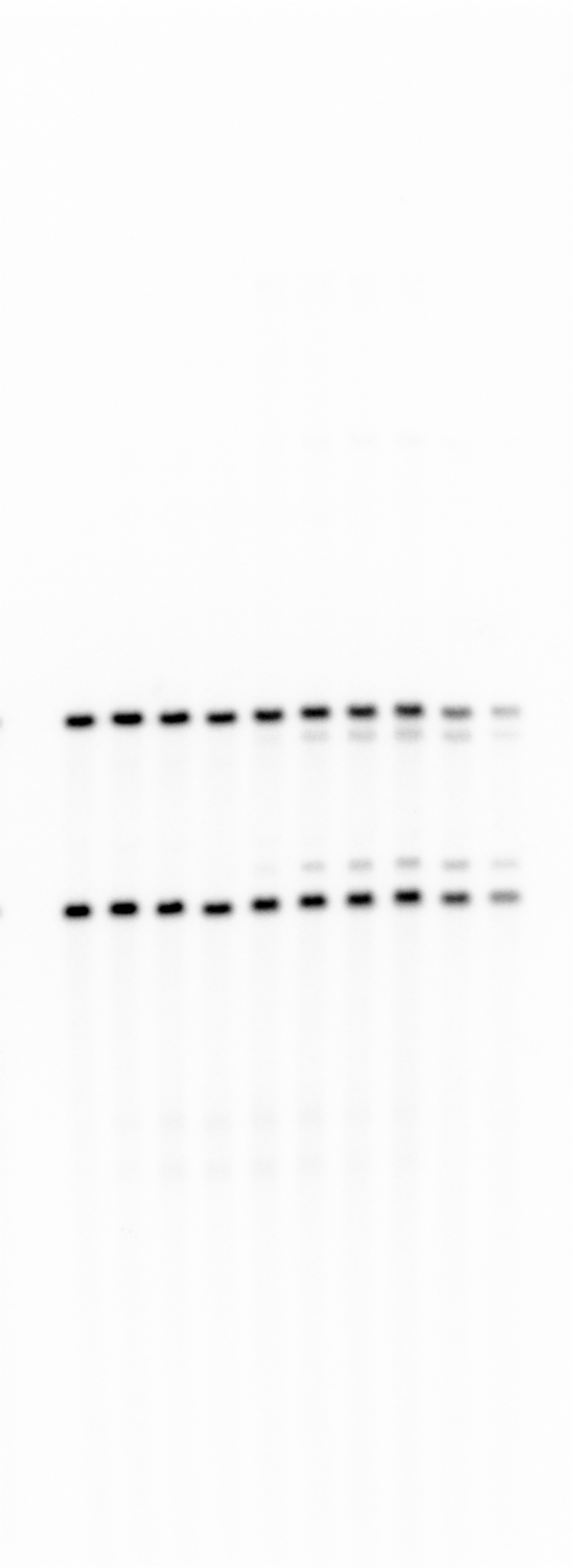

Supplement: Supplementary file 5 — Source data Fig. 6 [file 44318_2024_318_MOESM5_ESM.zip › SD Fig 6/6A/fun30D_1D gel.tif]

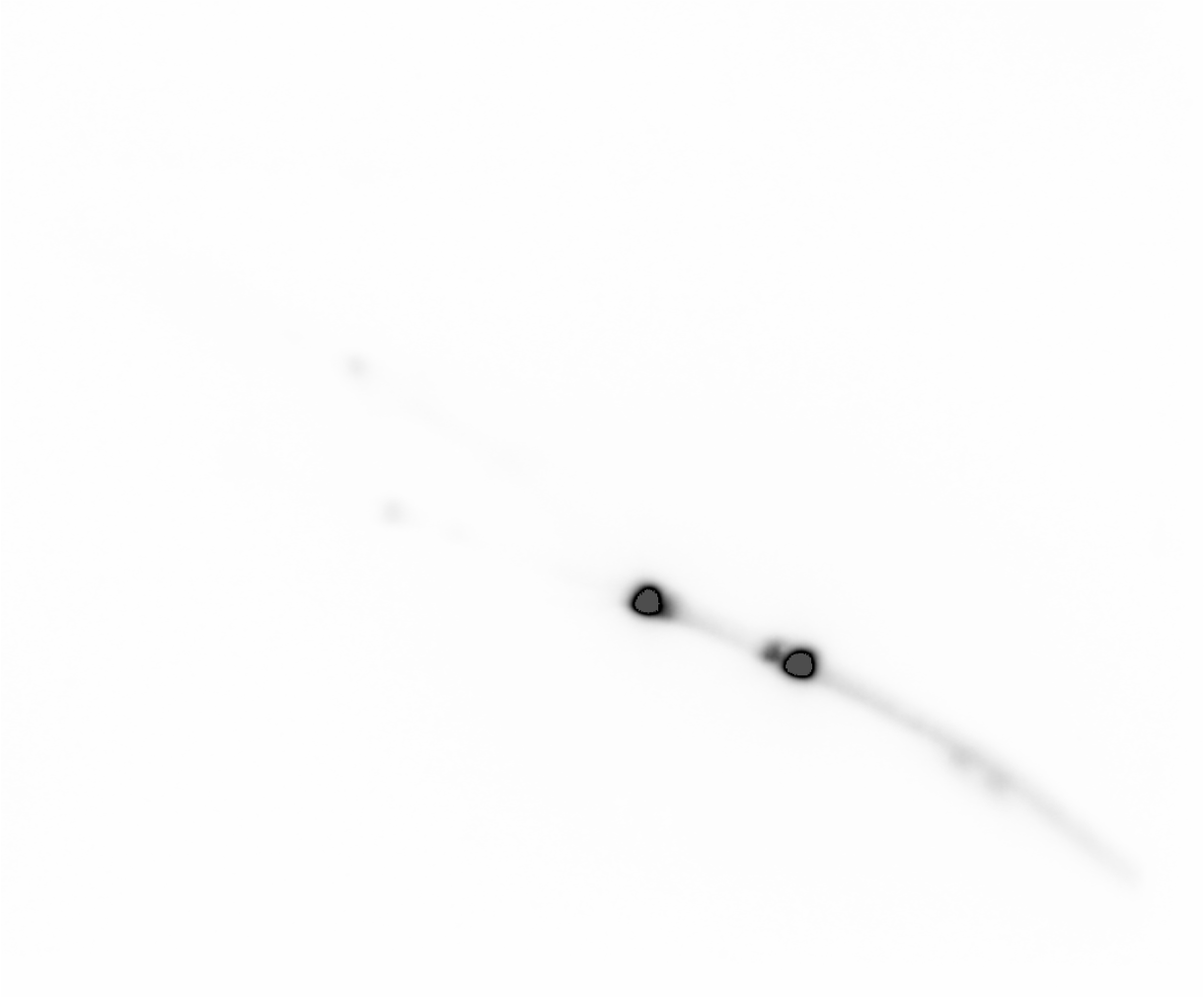

Supplement: Supplementary file 5 — Source data Fig. 6 [file 44318_2024_318_MOESM5_ESM.zip › SD Fig 6/6D/fun30D_2D gel.tif]

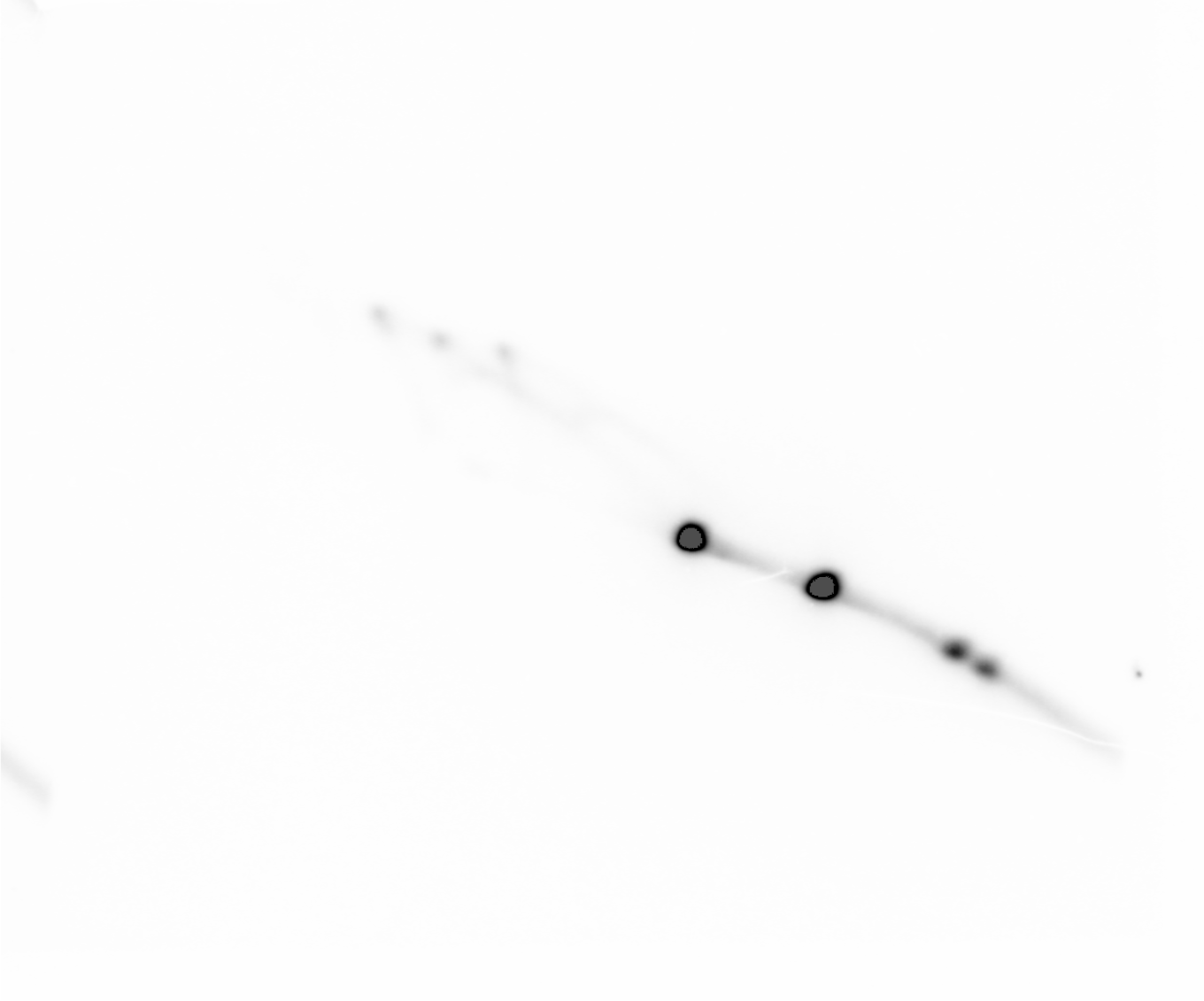

Supplement: Supplementary file 5 — Source data Fig. 6 [file 44318_2024_318_MOESM5_ESM.zip › SD Fig 6/6D/fun30D exo1-nd_2D gel.tif]

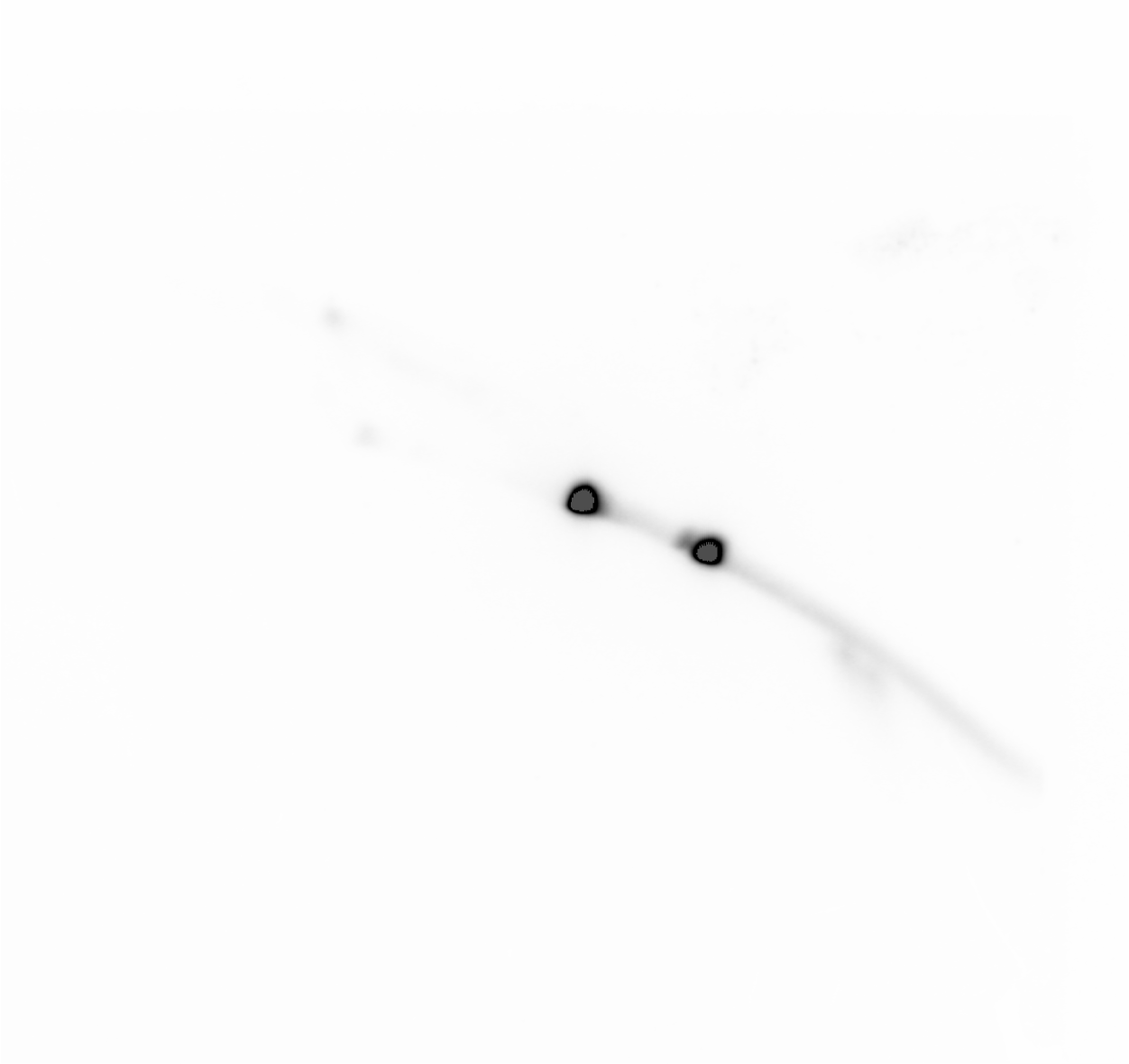

Supplement: Supplementary file 5 — Source data Fig. 6 [file 44318_2024_318_MOESM5_ESM.zip › SD Fig 6/6D/WT_2D gel.tif]

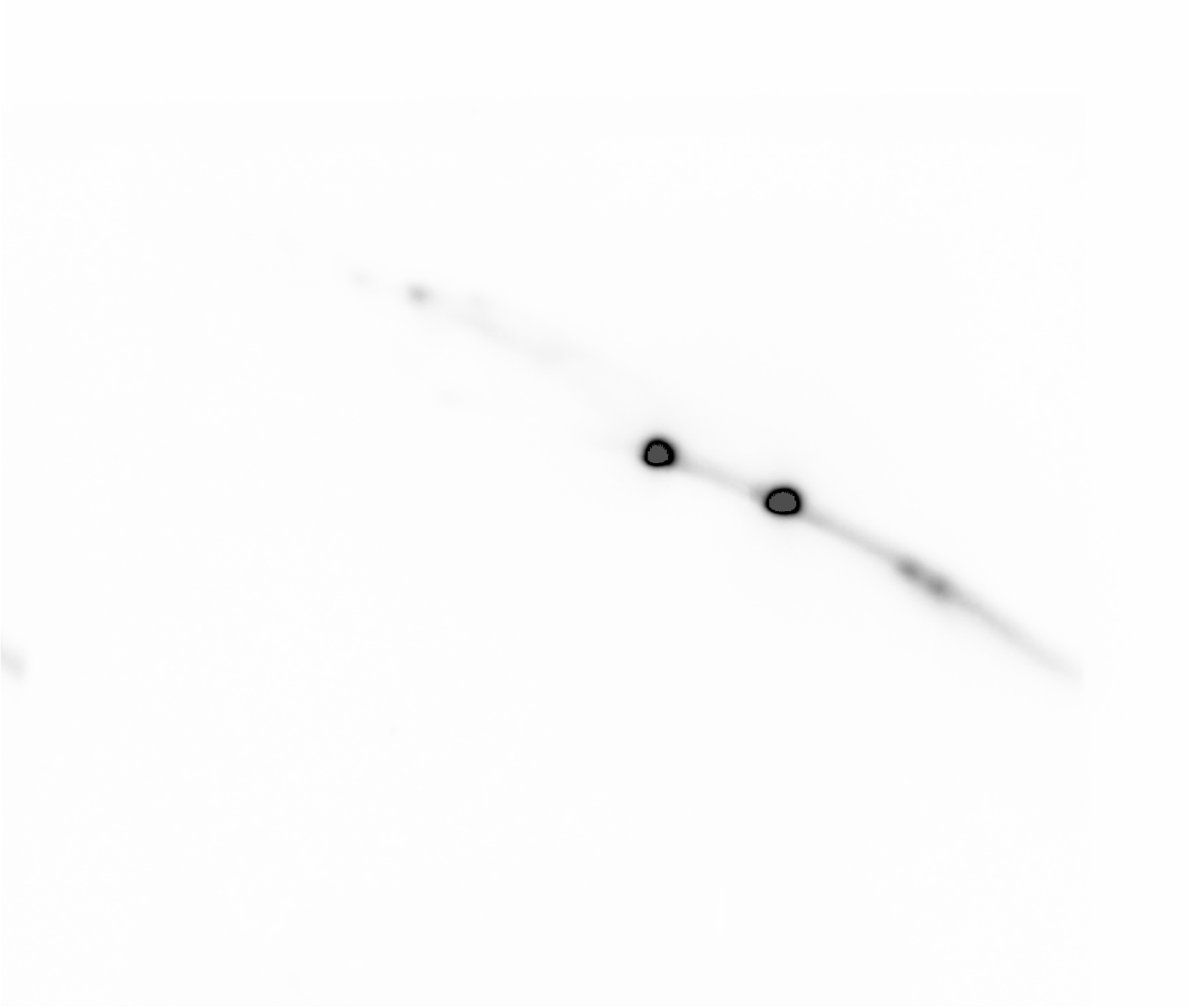

Supplement: Supplementary file 5 — Source data Fig. 6 [file 44318_2024_318_MOESM5_ESM.zip › SD Fig 6/6D/exo1-nd_2D gel.tif]

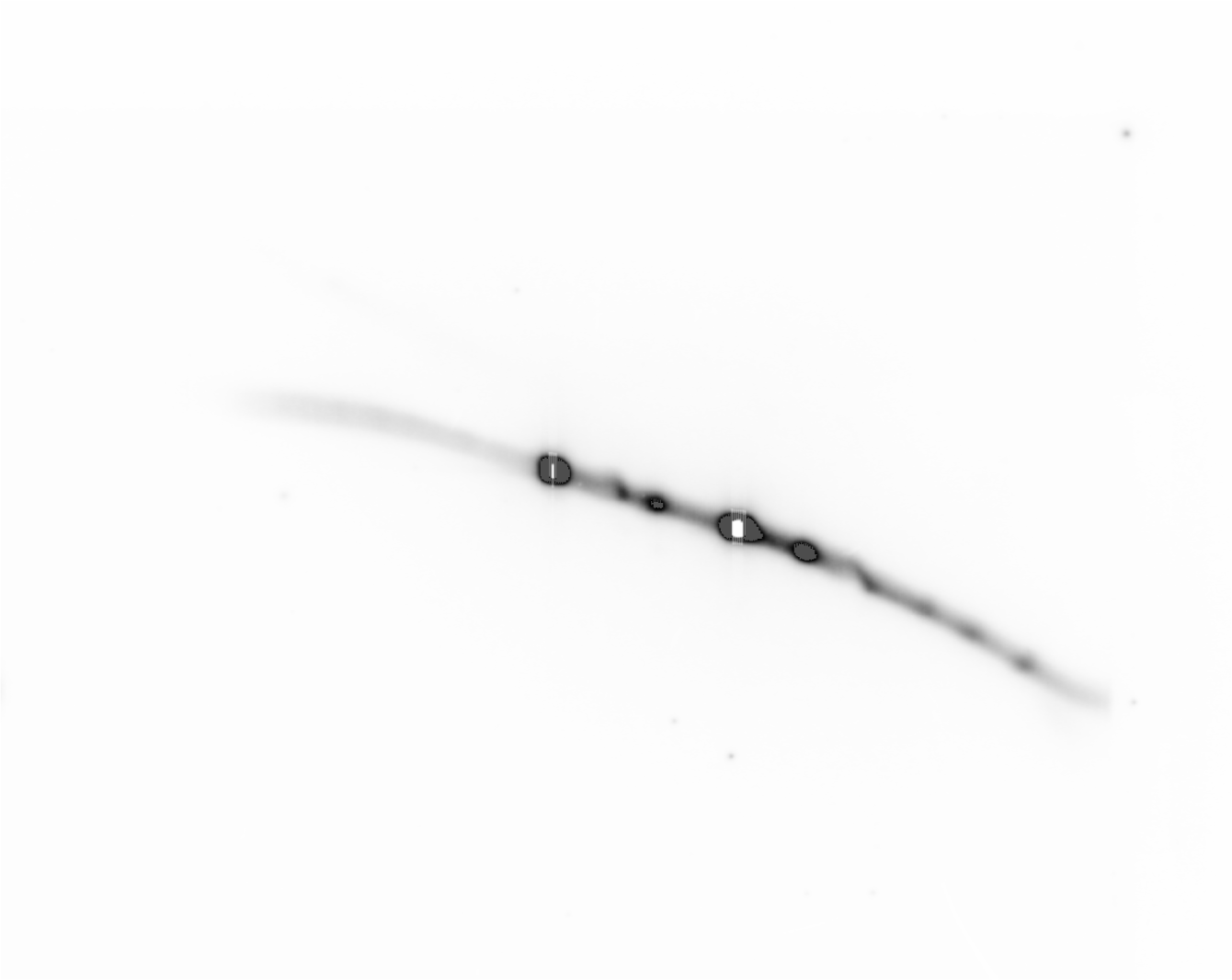

Supplement: Supplementary file 6 — Source data Fig. 7 [file 44318_2024_318_MOESM6_ESM.zip › SD Fig 7/7B/ERG1_WT_2D gel.tif]

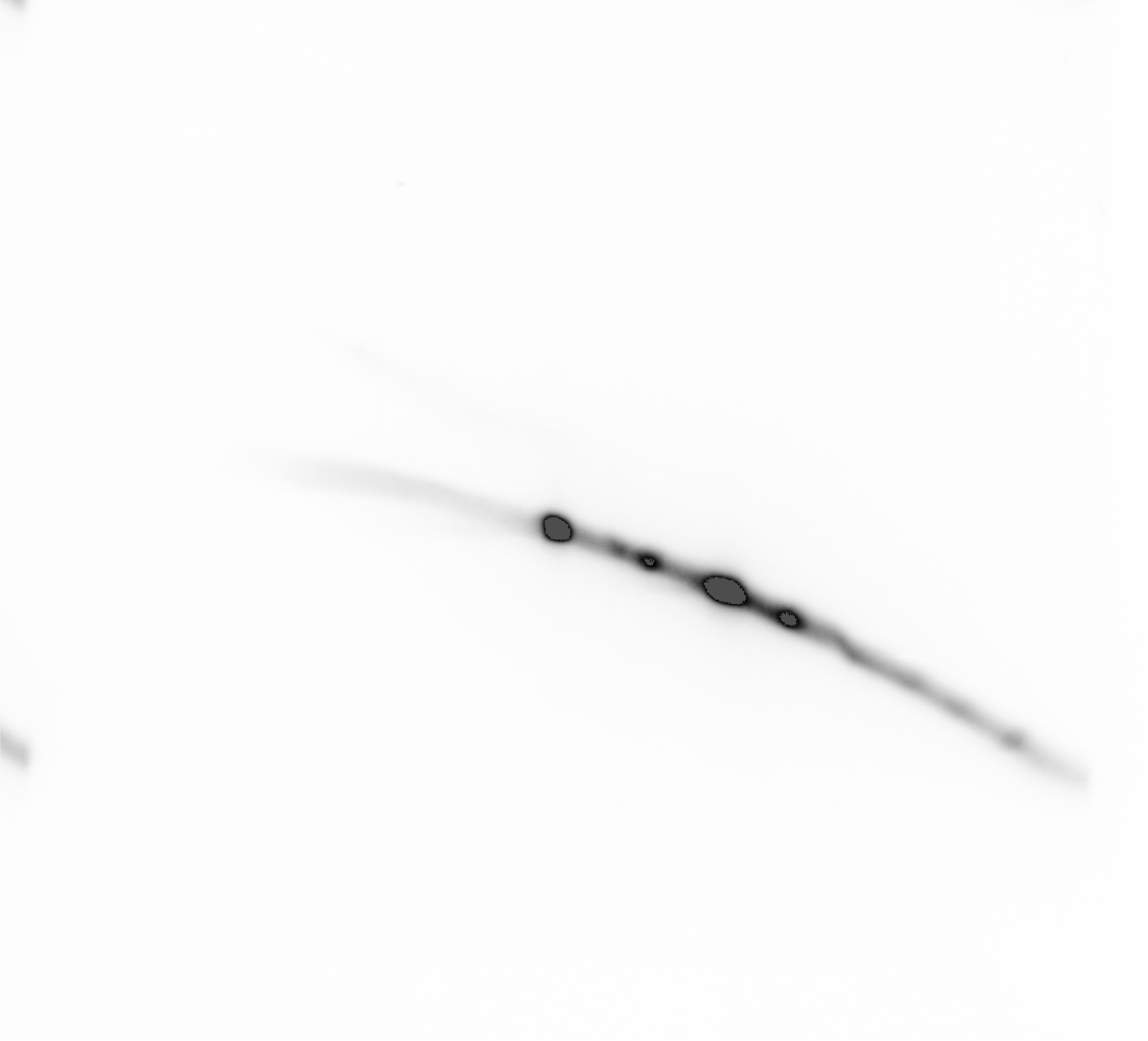

Supplement: Supplementary file 6 — Source data Fig. 7 [file 44318_2024_318_MOESM6_ESM.zip › SD Fig 7/7B/ERG1_fun30D_2D gel.tif]

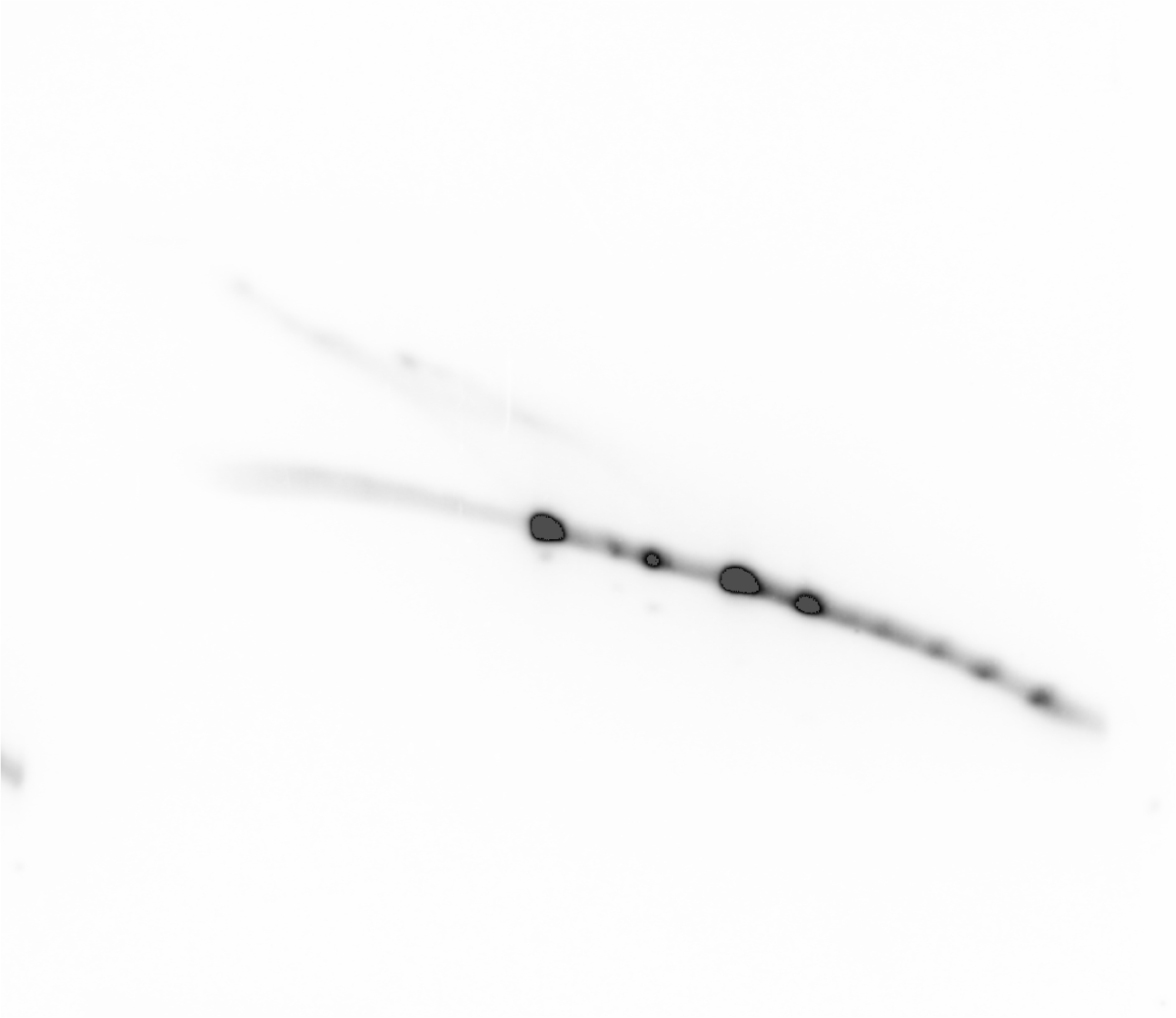

Supplement: Supplementary file 6 — Source data Fig. 7 [file 44318_2024_318_MOESM6_ESM.zip › SD Fig 7/7B/ERG1_fun30D exo1-nd_2D gel.tif]

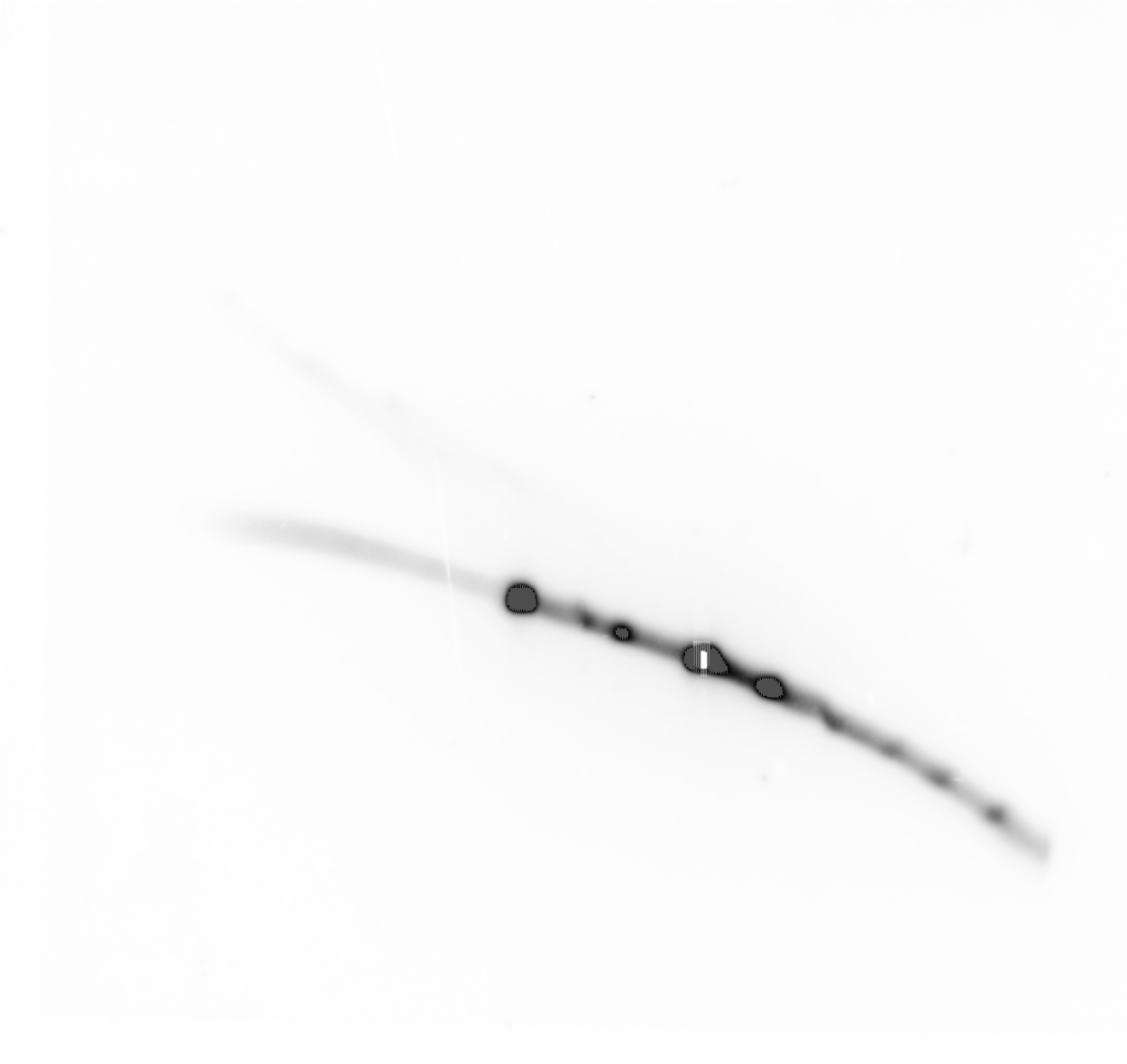

Supplement: Supplementary file 6 — Source data Fig. 7 [file 44318_2024_318_MOESM6_ESM.zip › SD Fig 7/7B/ERG1_exo1-nd_2D gel.tif]
