# Supplementary material for: Meiotic DNA break resection and recombination rely on chromatin remodeler Fun30
Source: EMBO J. 2024 Nov 29;44(1):200–24. doi: 10.1038/s44318-024-00318-8 (PMC11695836; doi:10.1038/s44318-024-00318-8)
Supplement: Supplementary file 7 — Expanded View Figures [file 44318_2024_318_MOESM7_ESM.pdf]

Expanded View Figures

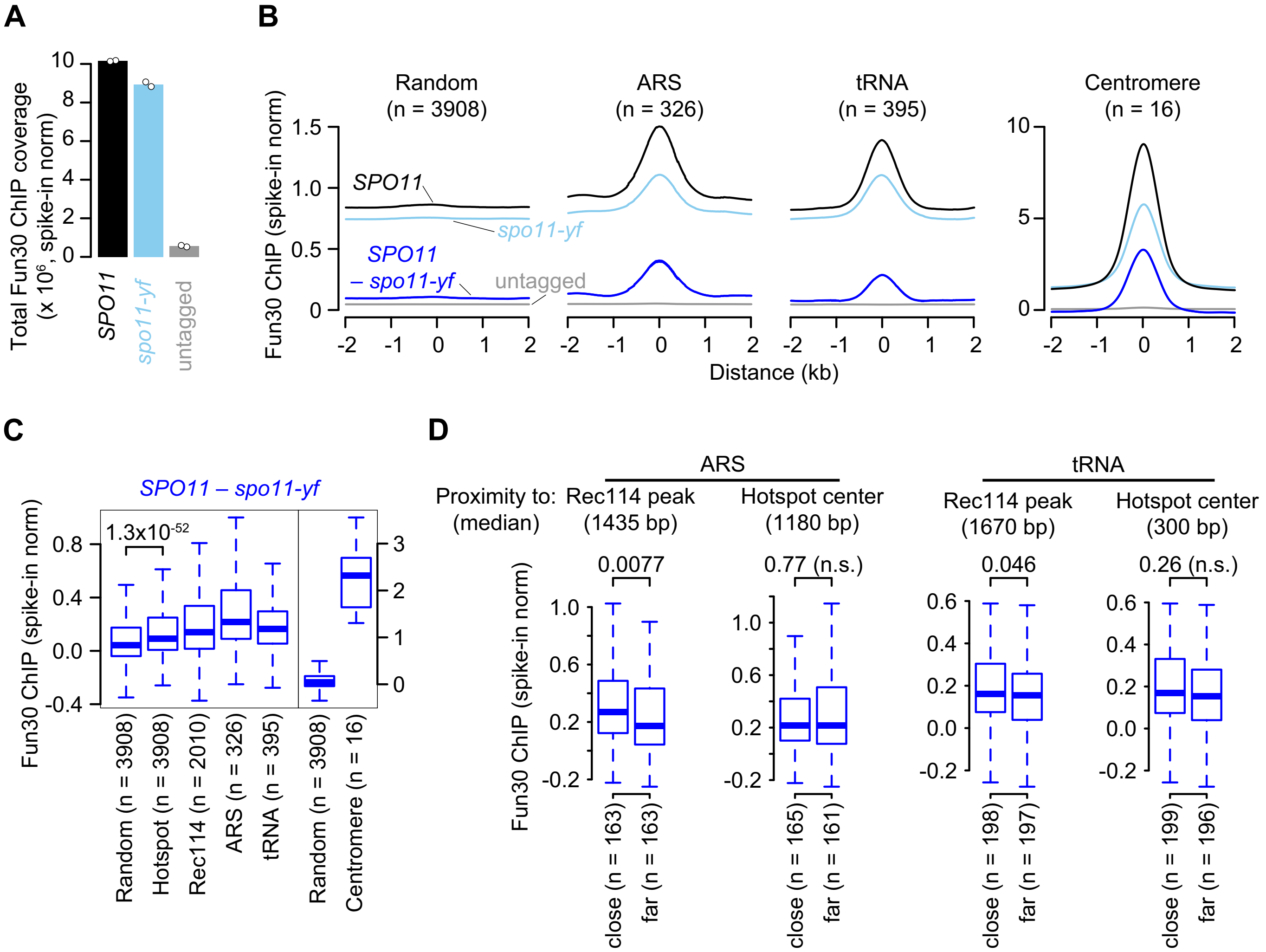

Figure EV1. DSB-dependent Fun30 enrichment.

(**A**) Total Fun30 ChIP-seq coverage normalized to the spike-in control. Bars are the means from two biological replicates; open circles show the individual values for each replicate. (**B**) Average Fun30 ChIP-seq signals around ARS, tRNA, and centromere. The random sites here and in (**C**) are the same as in Fig. 3B. (**C**) DSB-dependent Fun30 enrichment. Box plots summarize the distributions across all of the indicated elements from (**B**) and Fig. 3B for Fun30 ChIP-seq signal summed in 1-kb windows. Note the different y-axis scales for left and right parts of the plot. In all box plots, thick horizontal bars denote medians, box edges mark the upper and lower quartiles, and whiskers indicate values within 1.5-fold of the interquartile range. Outliers are not shown. Here and in (**D**), numbers above brackets indicate p values of two-sided Wilcoxon tests. (**D**) ARS and tRNAs that are closer to Rec114 binding sites tend to exhibit higher DSB-dependent Fun30 ChIP-seq signals. The ARS and tRNA regions from (**B**, **C**) were subdivided into two groups based on the distance to the nearest Rec114 peak or hotspot center: "close" indicates elements less than the median distance away and "far" indicates the rest. Proximity to Rec114 peaks was associated with a significantly higher DSB-dependent Fun30 ChIP-seq signal, whereas proximity to hotspots showed no such pattern. These results suggest that at least some of the DSB-dependent recruitment of Fun30 to ARS or tRNA genes is a consequence of fortuitous proximity or overlap of (some of) these elements with Rec114 ChIP peaks.

 © The Author(s)

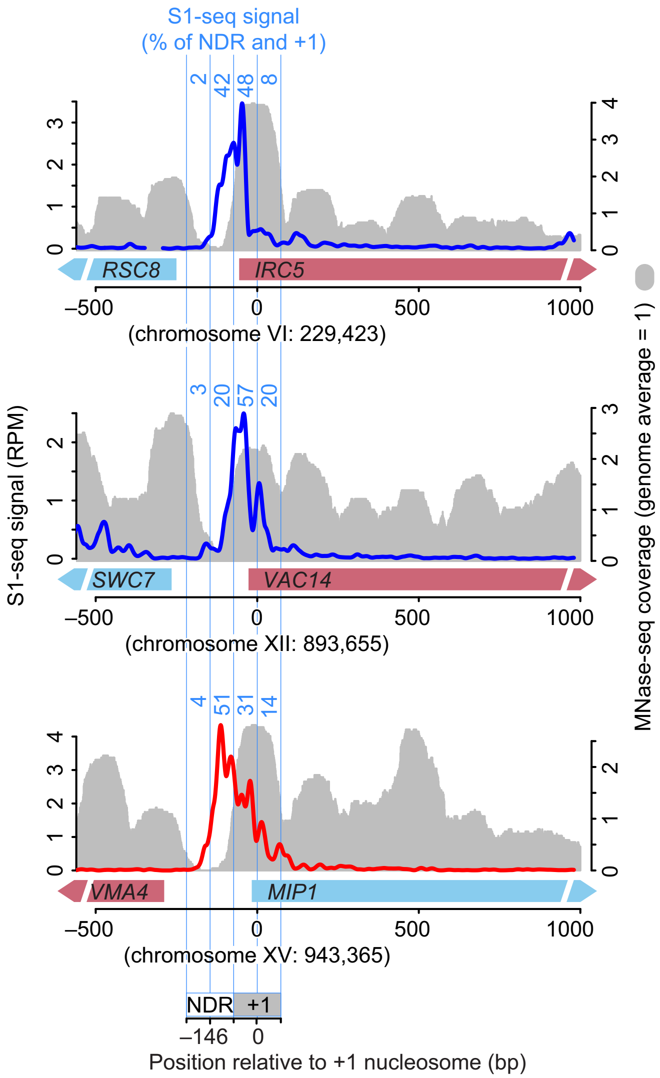

Figure EV2. MRX/Sae2 nicks within the +1 nucleosome.

Examples of resection endpoint distributions in *exo1-nd fun30Δ* at three representative loci that contributed to the average shown in Fig. 4B. S1-seq signals (41-bp smoothed) from the top (blue) or bottom (red) strand are shown, dependent on the orientation of the gene where the +1 nucleosome is located. Numbers in light blue indicate the percentages of S1-seq signal in the four windows spanning the +1 nucleosome and NDR (see legend to Fig. 4B).

© The Author(s)

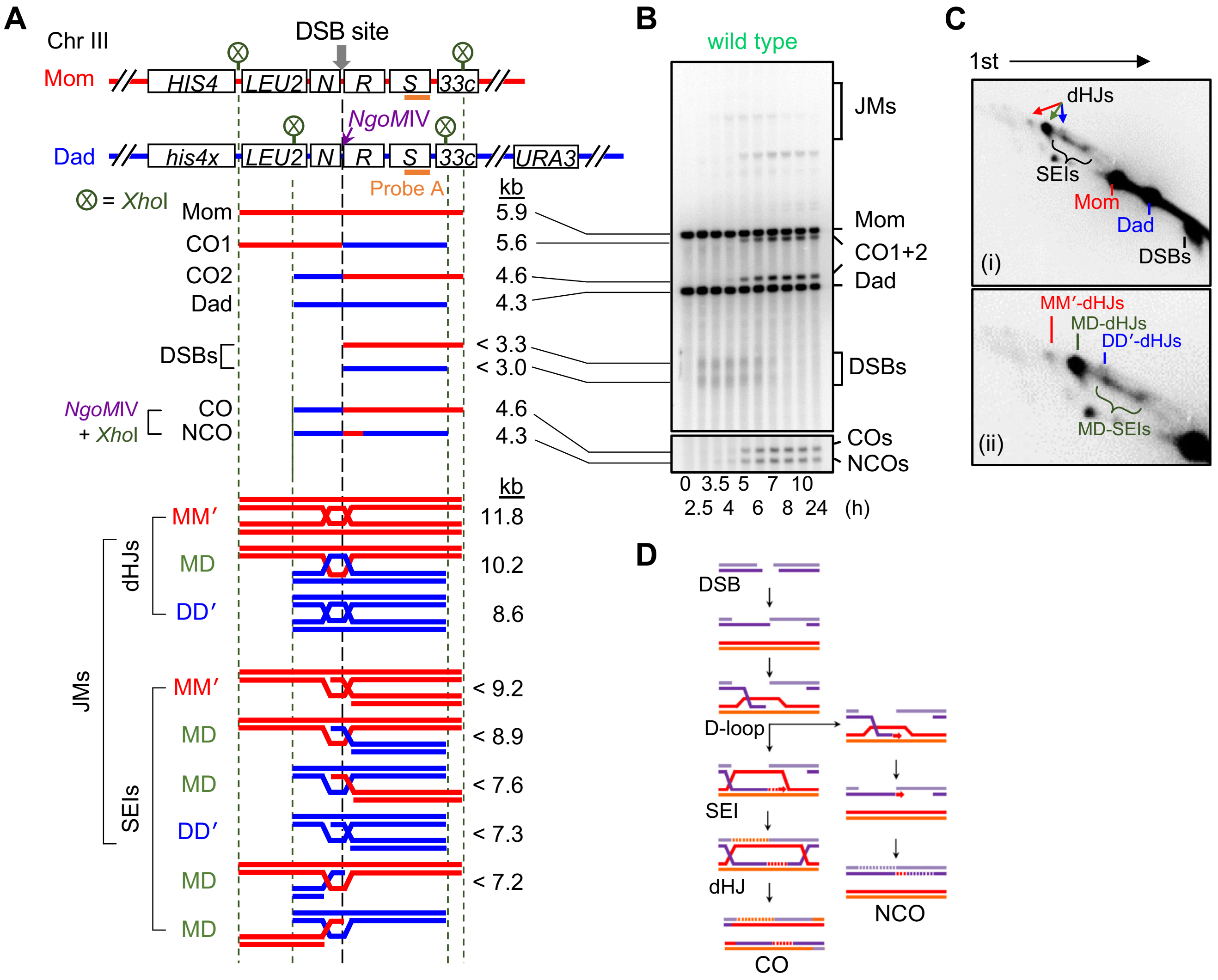

Figure EV3. Physical assay detecting recombination intermediates at the *HIS4LEU2* hotspot.

(**A**) Physical map of the *HIS4LEU2* locus showing diagnostic *Xho*I restriction enzyme sites and the position of Southern blot probe A. "Mom" and "Dad" indicate the two parental versions of the locus; COs, crossovers; NCOs, noncrossovers; MM′ IS-dHJ, intersister double-Holliday junction; MD IH-dHJ, interhomolog double-Holliday junction; DD′ IS-dHJ, intersister double-Holliday junction; SEIs, single-end invasions. Positions of *Xho*I sites are indicated as circled Xs. (**B**) Example one-dimensional gel analysis showing parental signals, DSBs, COs, NCOs, and joint molecules (JMs). The Southern blot images are reproduced from Fig. 6A,F. (**C**) Example two-dimensional gel displaying parental signals and recombination intermediates. Green arrow or text indicate interhomolog species; red and blue arrows and text indicate intersister species. (**D**) Key steps in crossover and noncrossover formation during meiosis.

 © The Author(s)

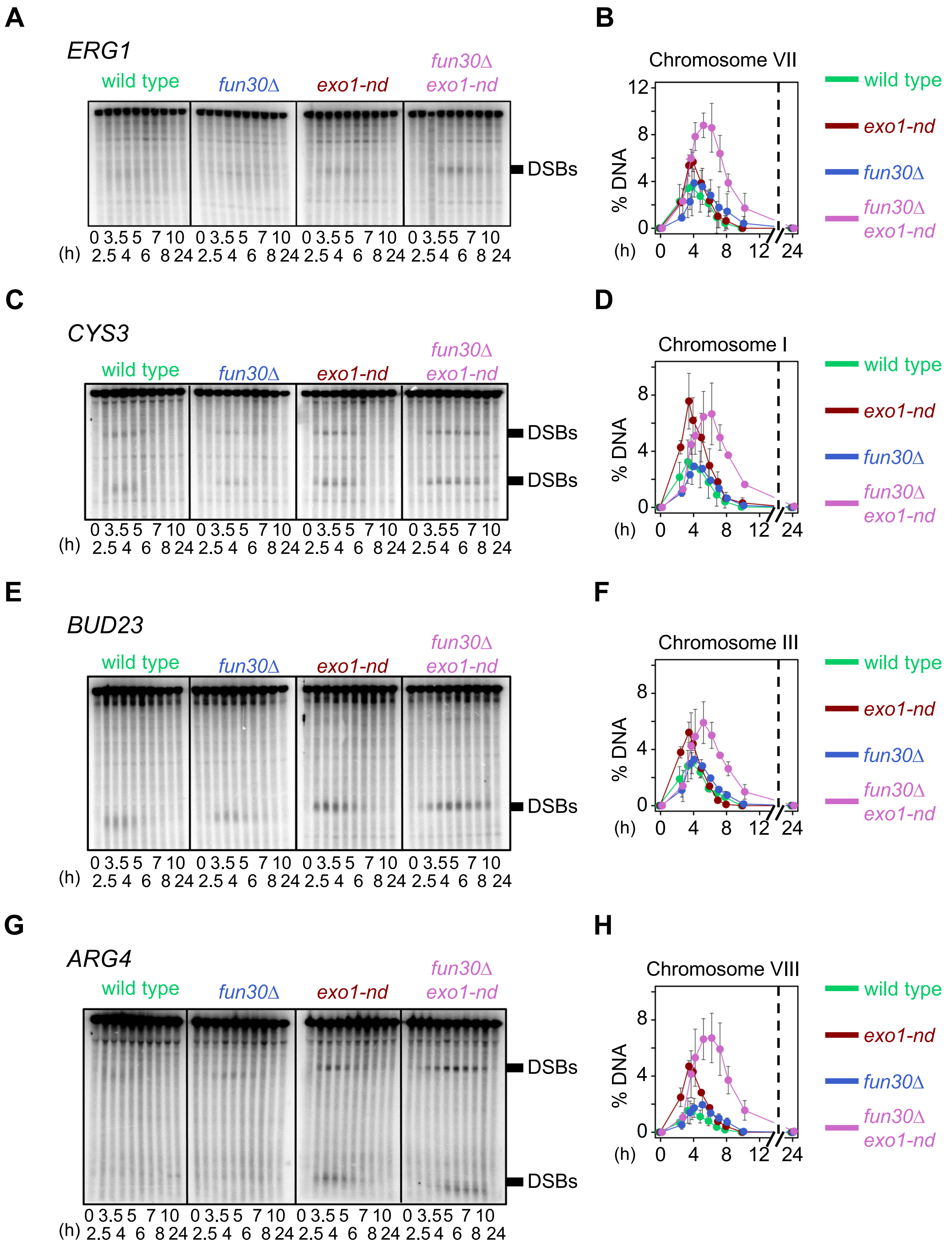
A
ERG1
wild type
fun30Δ
exo1-nd
fun30Δ exo1-nd
DSBs
0 3.5 5 7 10
(h) 2.5 4 6 8 24
B
Chromosome VII
% DNA
12
8
4
0
(h) 0 4 8 12 24
wild type
exo1-nd
fun30Δ
fun30Δ exo1-nd
C
CYS3
wild type
fun30Δ
exo1-nd
fun30Δ exo1-nd
DSBs
DSBs
D
Chromosome I
% DNA
E
BUD23
wild type
fun30Δ
exo1-nd
fun30Δ exo1-nd
DSBs
F
Chromosome III
% DNA
G
ARG4
wild type
fun30Δ
exo1-nd
fun30Δ exo1-nd
DSBs
DSBs
H
Chromosome VIII
% DNA

Figure EV4. DSBs formation at various natural hotspots.

(**A–H**) Representative one-dimensional gel analyses of DSBs and corresponding quantification at the *ERG1* (**A**, **B**), *CYS3* (**C**, **D**), *BUD23* (**E**, **F**), and *ARG4* (**G**, **H**) hotspots. Error bars indicate mean ± SD for three independent cultures.

© The Author(s)

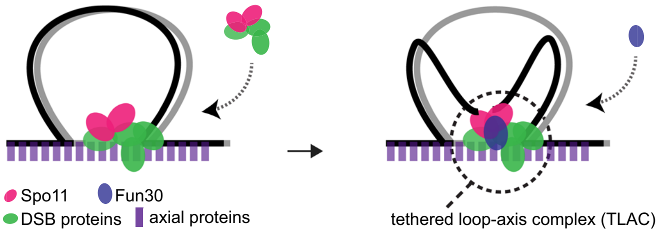

Figure EV5. Schematic representation of DSB-dependent Fun30 recruitment in the tethered loop-axis complex.

A model proposed based on findings in this study. In response to Spo11 cleaving DNA within the TLAC, Fun30 is recruited to the DSB ends and remodels the nucleosomes.

© The Author(s)
